# Supplementary material for: A motor neuron disease-associated mutation produces non-glycosylated Seipin that induces ER stress and apoptosis by inactivating SERCA2b
Source: eLife. 2022 Nov 29;11:e74805. doi: 10.7554/eLife.74805 (PMC9708084; doi:10.7554/eLife.74805)
Supplement: Figure 1—source data 1. [file elife-74805-fig1-data1.zip › Fig. 1-source data 1/Fig. 1-source data.pdf]

Fig.1-source data

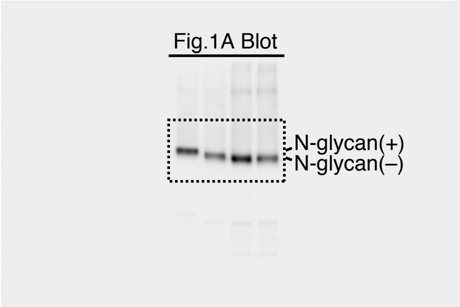

Mouse Anti-Myc Direct

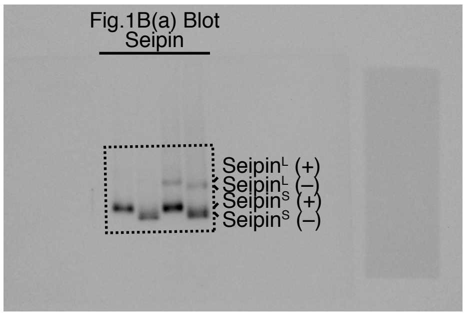

Rabbit Anti-Seipin

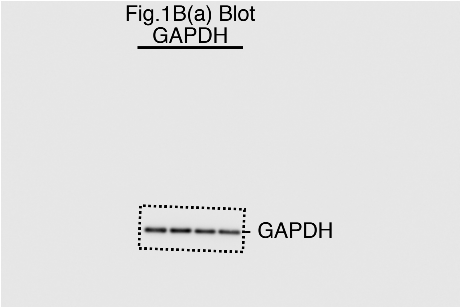

Mouse Anti-GAPDH Direct

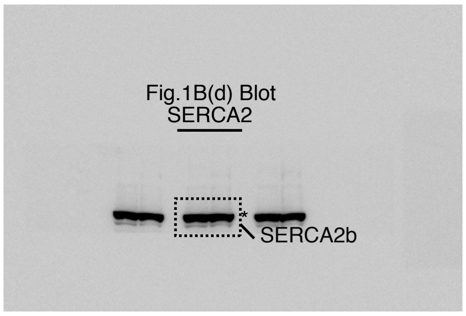

Mouse Anti-SERCA2

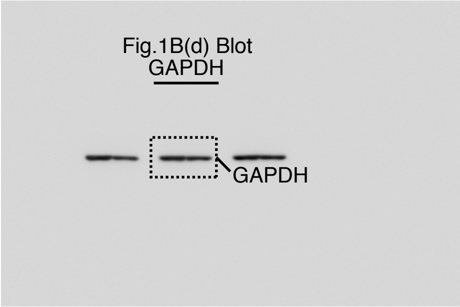

Mouse Anti-GAPDH Direct

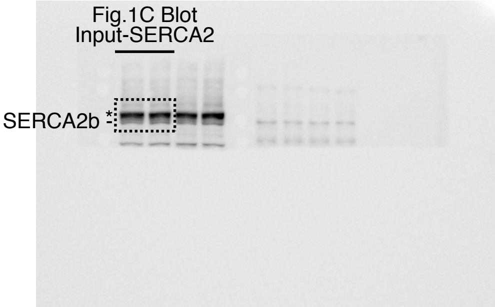

Mouse Anti-SERCA2

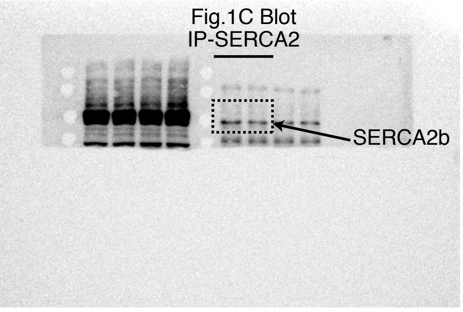

Mouse Anti-SERCA2

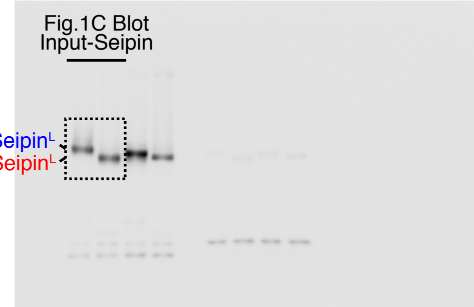

Mouse Anti-Myc Direct

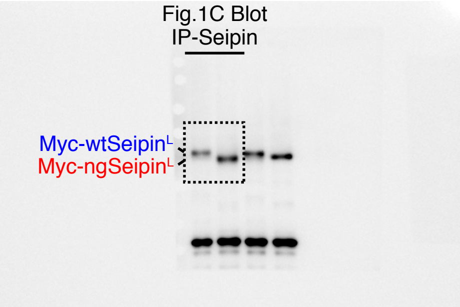

Mouse Anti-Myc Direct

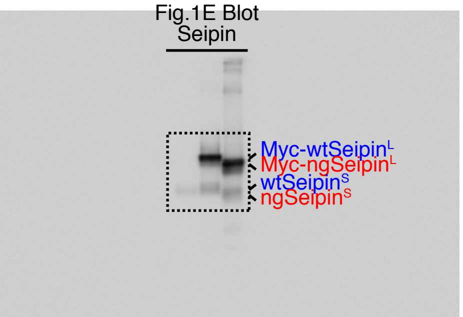

Rabbit Anti-Seipin

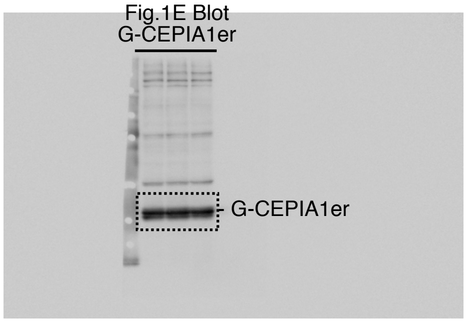

Rabbit Anti-GFP

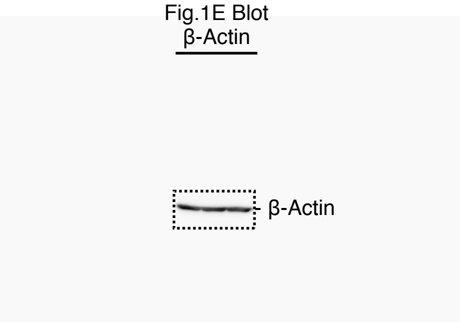

Mouse Anti-β-Actin
