# Supplementary figures and images for: A motor neuron disease-associated mutation produces non-glycosylated Seipin that induces ER stress and apoptosis by inactivating SERCA2b
Source: eLife. 2022 Nov 29;11:e74805. doi: 10.7554/eLife.74805 (PMC9708084; doi:10.7554/eLife.74805)

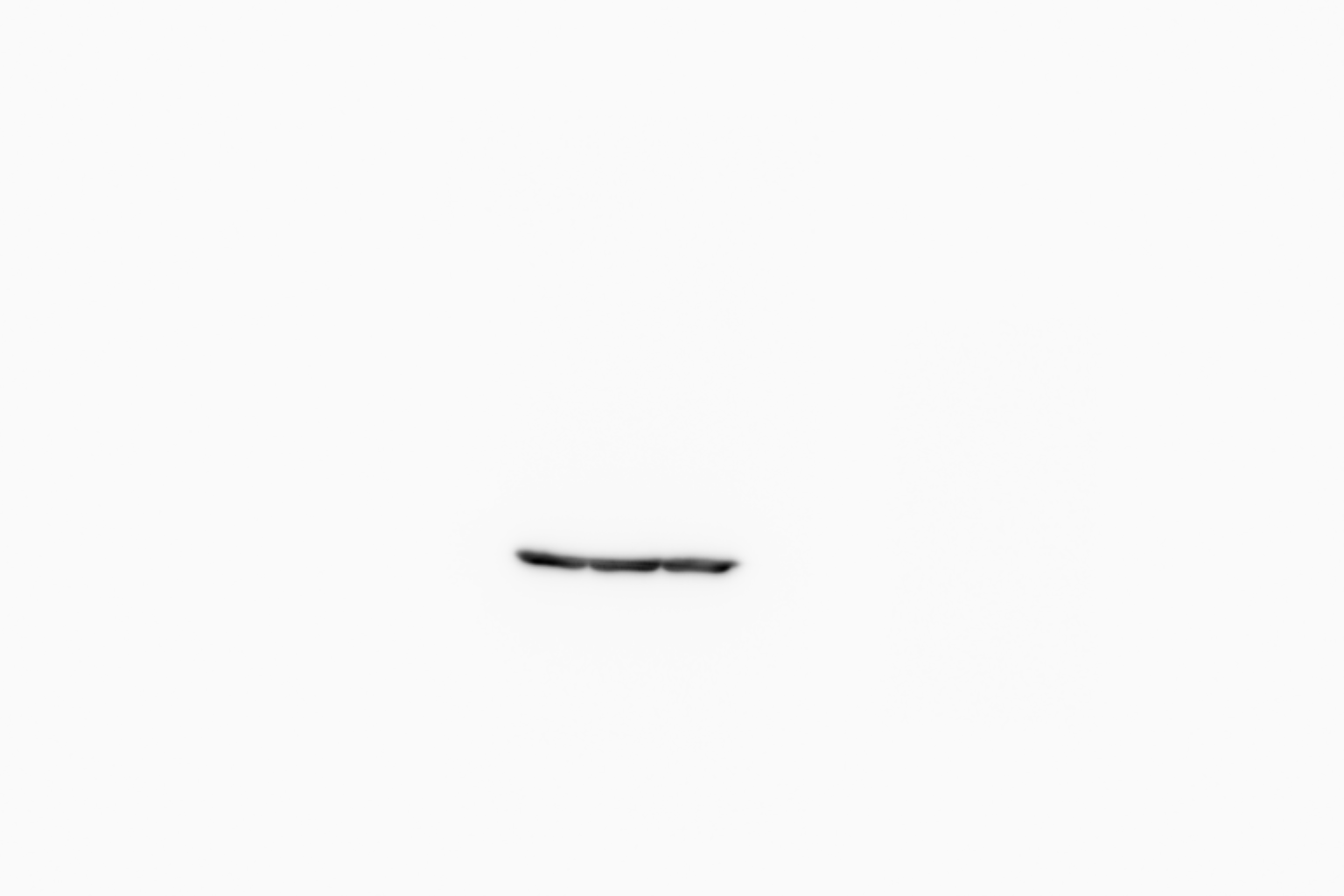

Supplement: Figure 1—source data 1. [file elife-74805-fig1-data1.zip › Fig. 1-source data 1/Fig. 1E Blot b-Actin .tif]

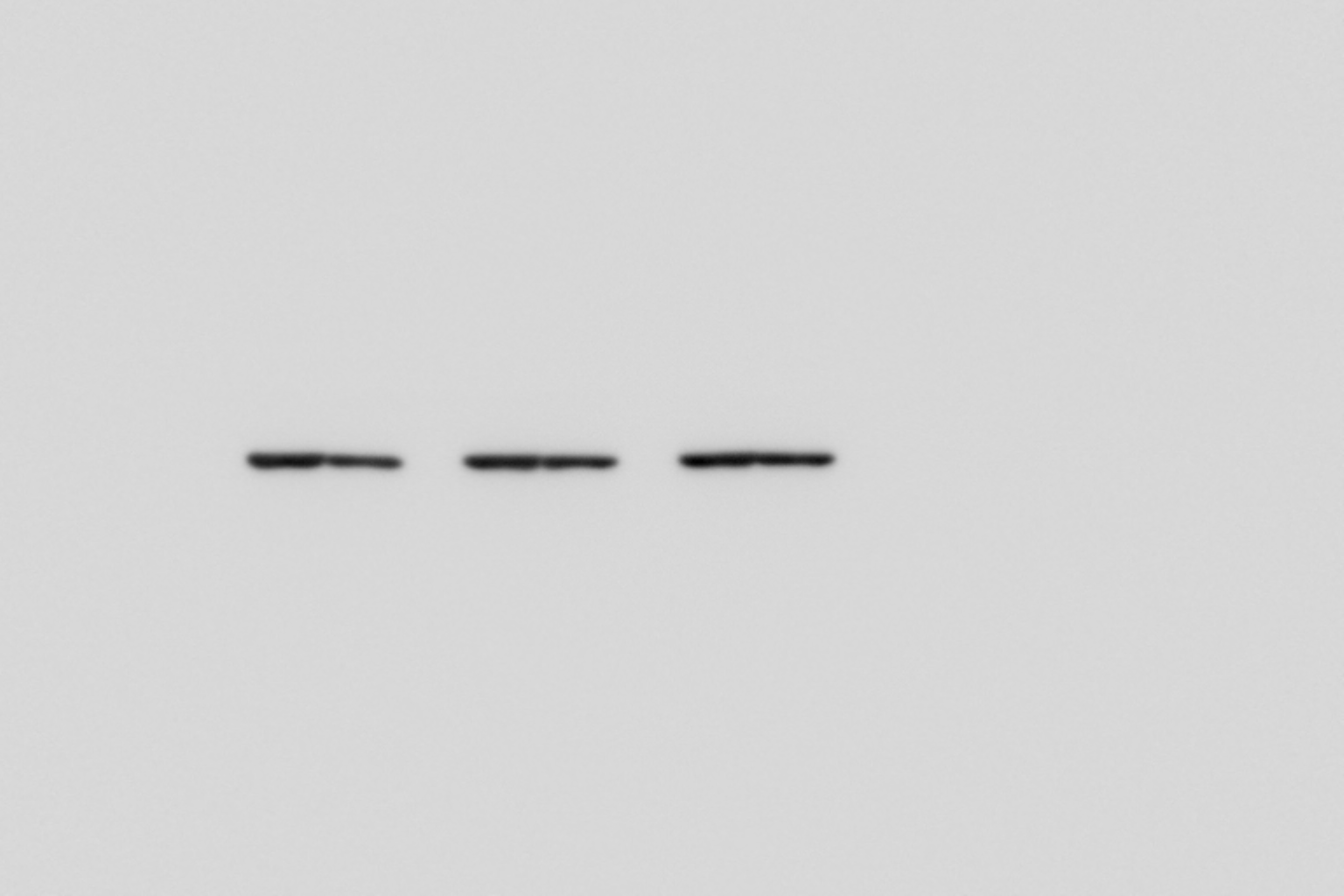

Supplement: Figure 1—source data 1. [file elife-74805-fig1-data1.zip › Fig. 1-source data 1/Fig. 1B(d) Blot GAPDH.tif]

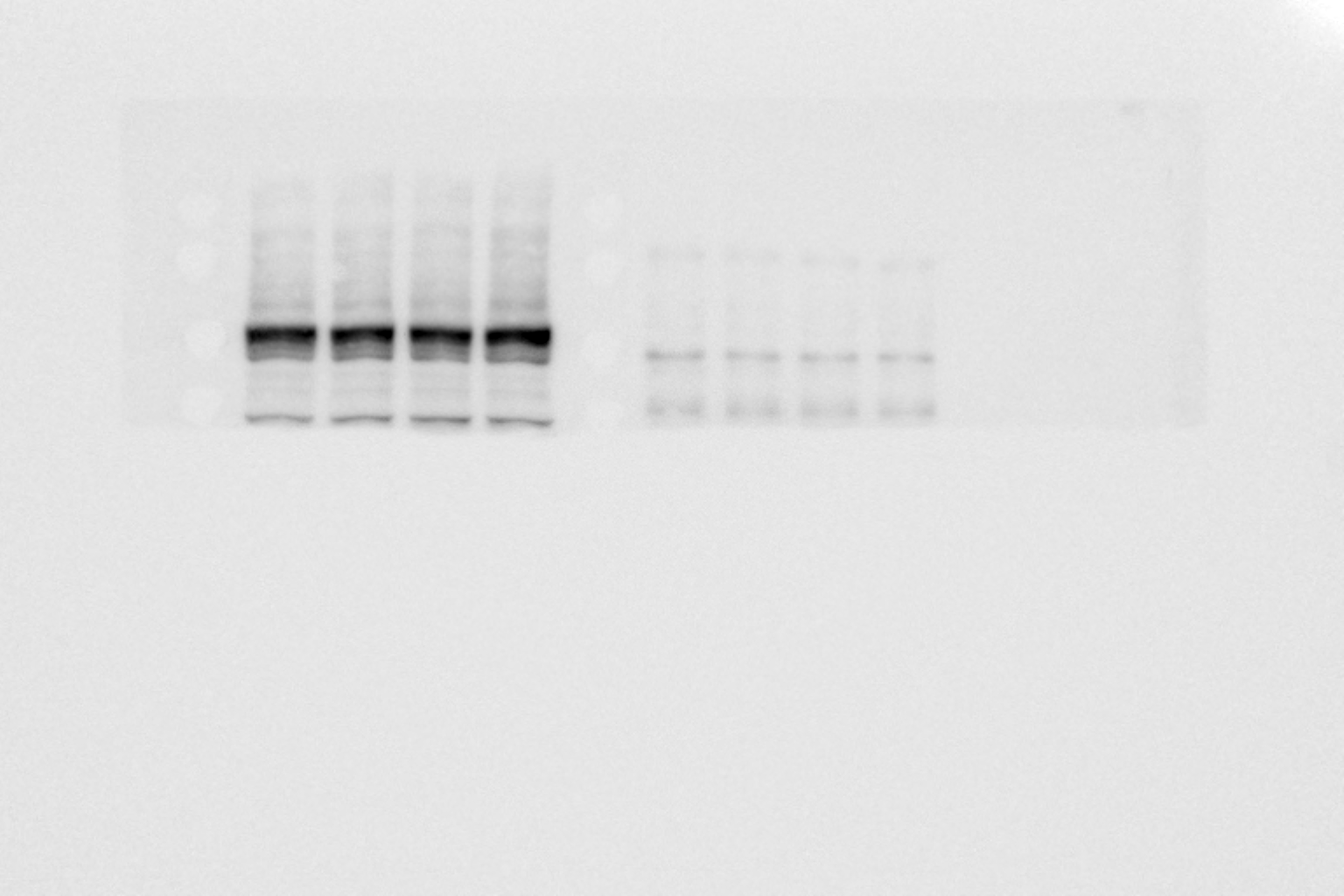

Supplement: Figure 1—source data 1. [file elife-74805-fig1-data1.zip › Fig. 1-source data 1/Fig. 1C Blot Input-SERCA2.tif]

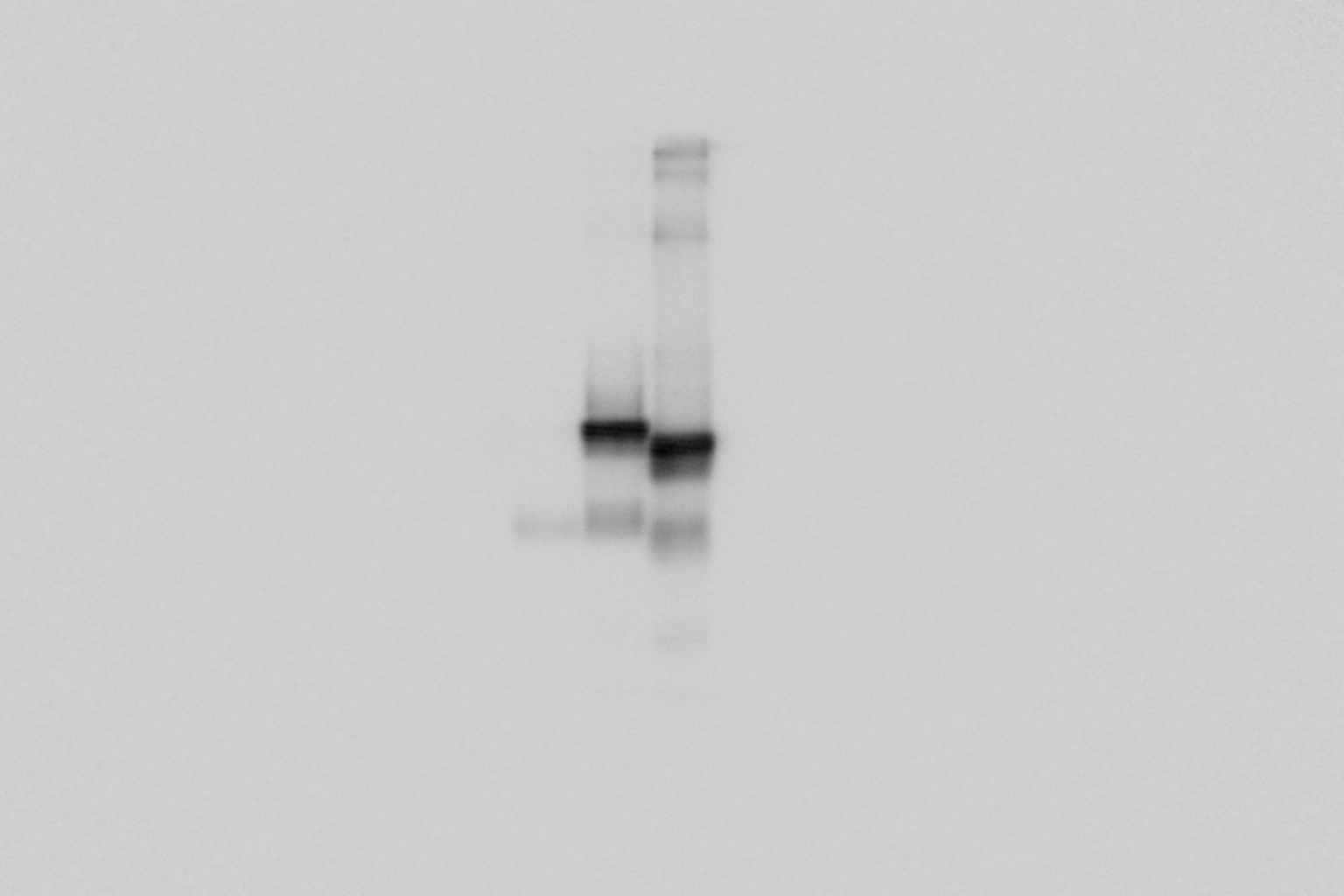

Supplement: Figure 1—source data 1. [file elife-74805-fig1-data1.zip › Fig. 1-source data 1/Fig. 1E Blot Seipin.tif]

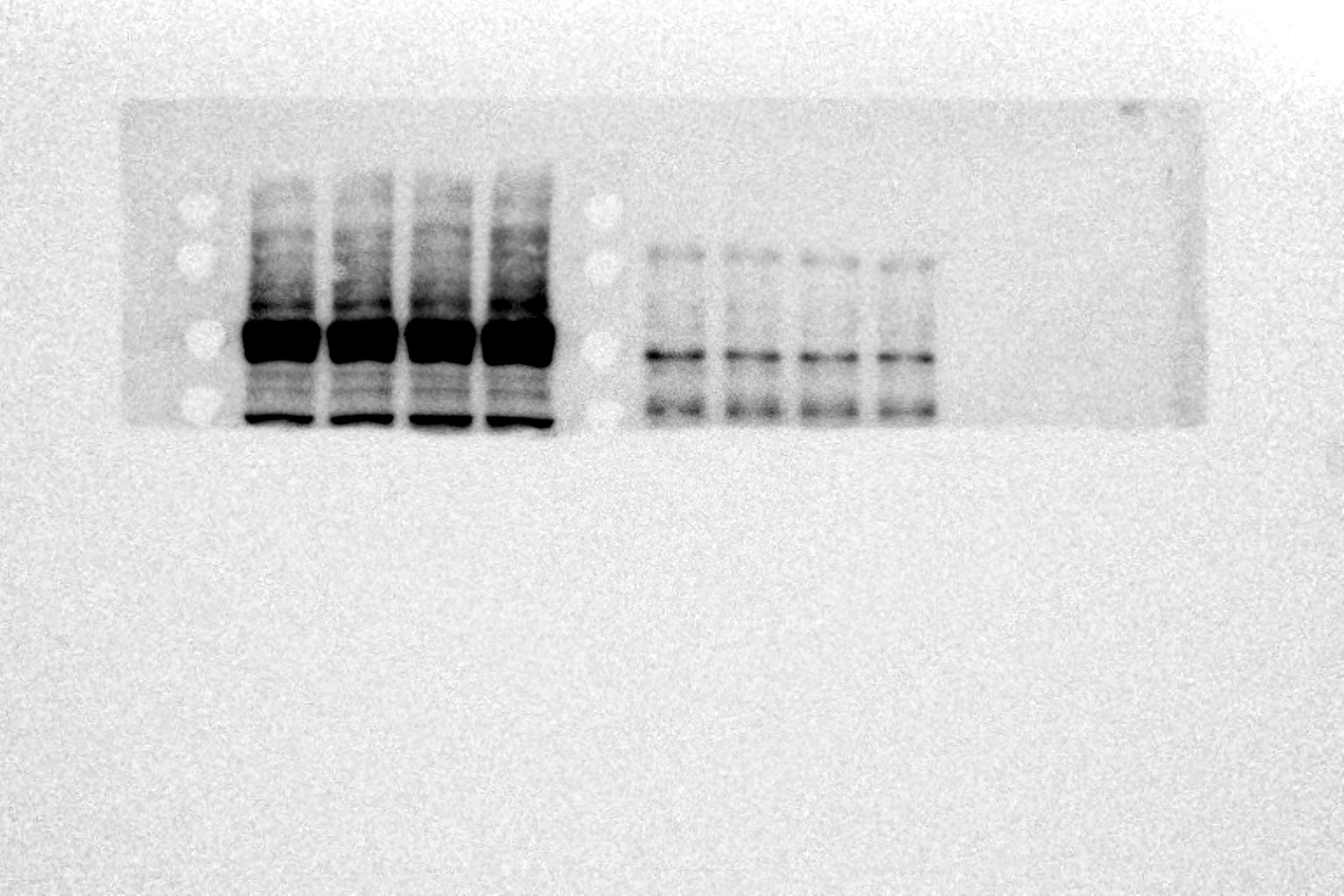

Supplement: Figure 1—source data 1. [file elife-74805-fig1-data1.zip › Fig. 1-source data 1/Fig. 1C Blot IP-SERCA2 .tif]

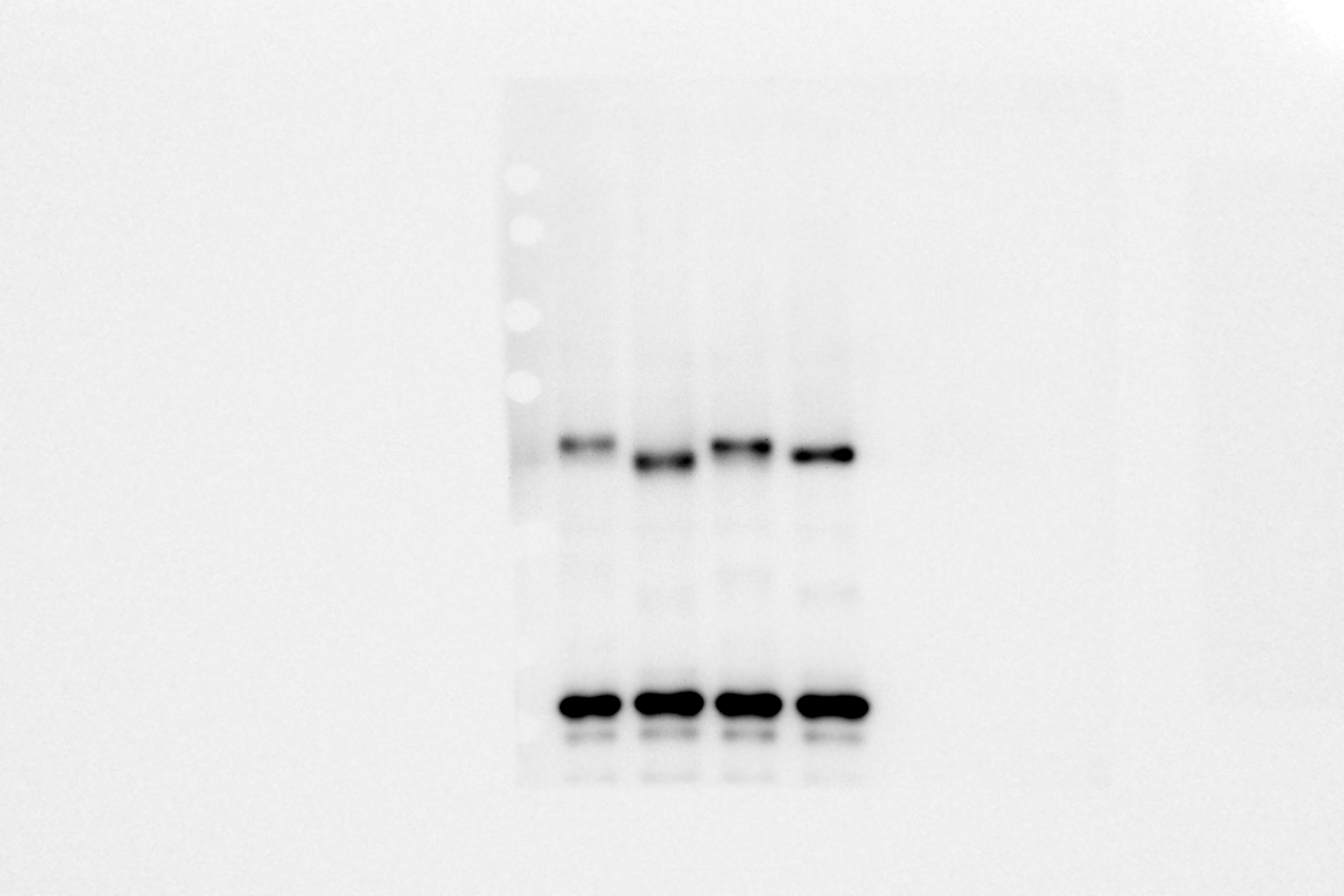

Supplement: Figure 1—source data 1. [file elife-74805-fig1-data1.zip › Fig. 1-source data 1/Fig. 1C Blot IP-Seipin.tif]

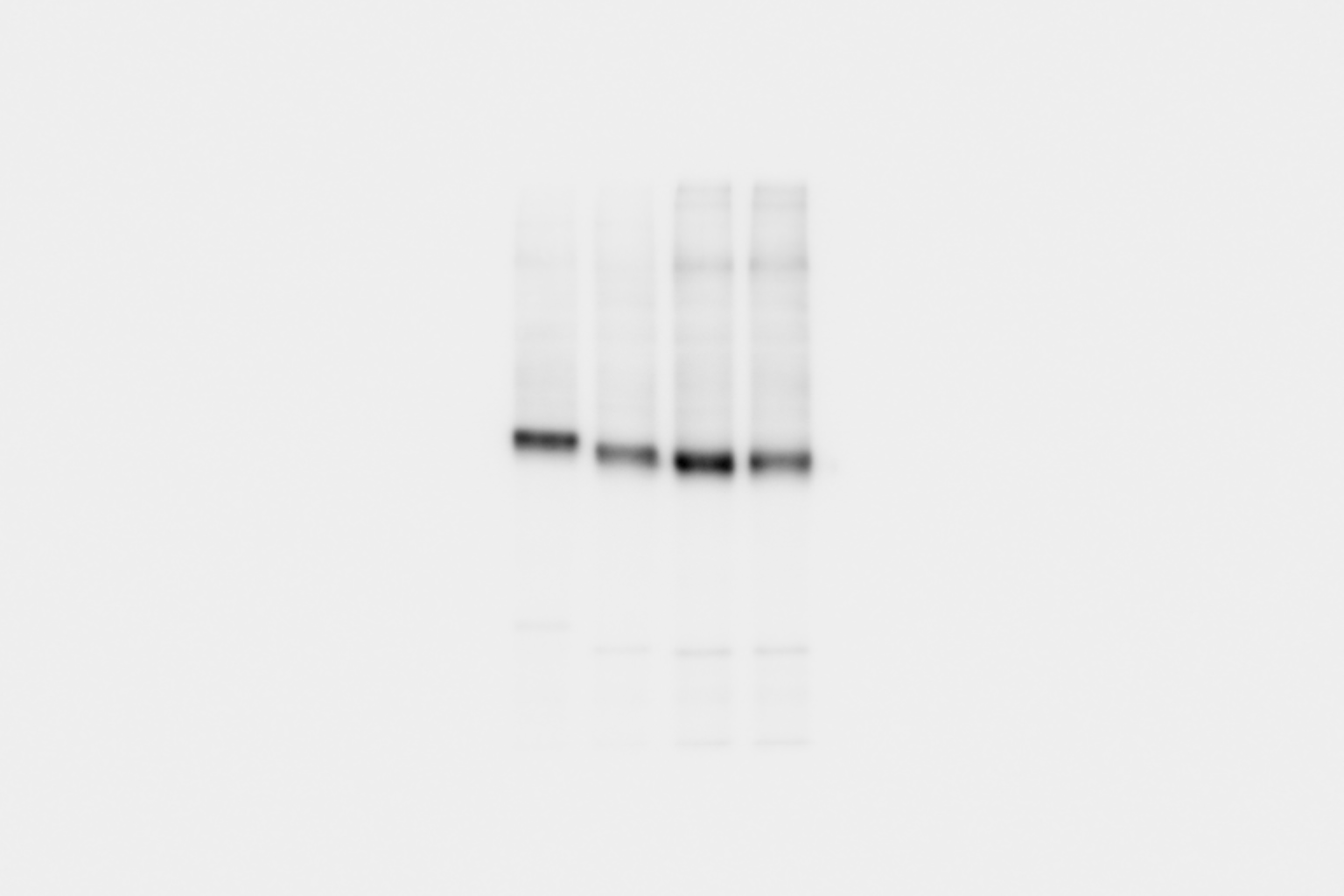

Supplement: Figure 1—source data 1. [file elife-74805-fig1-data1.zip › Fig. 1-source data 1/Fig. 1A Blot.tif]

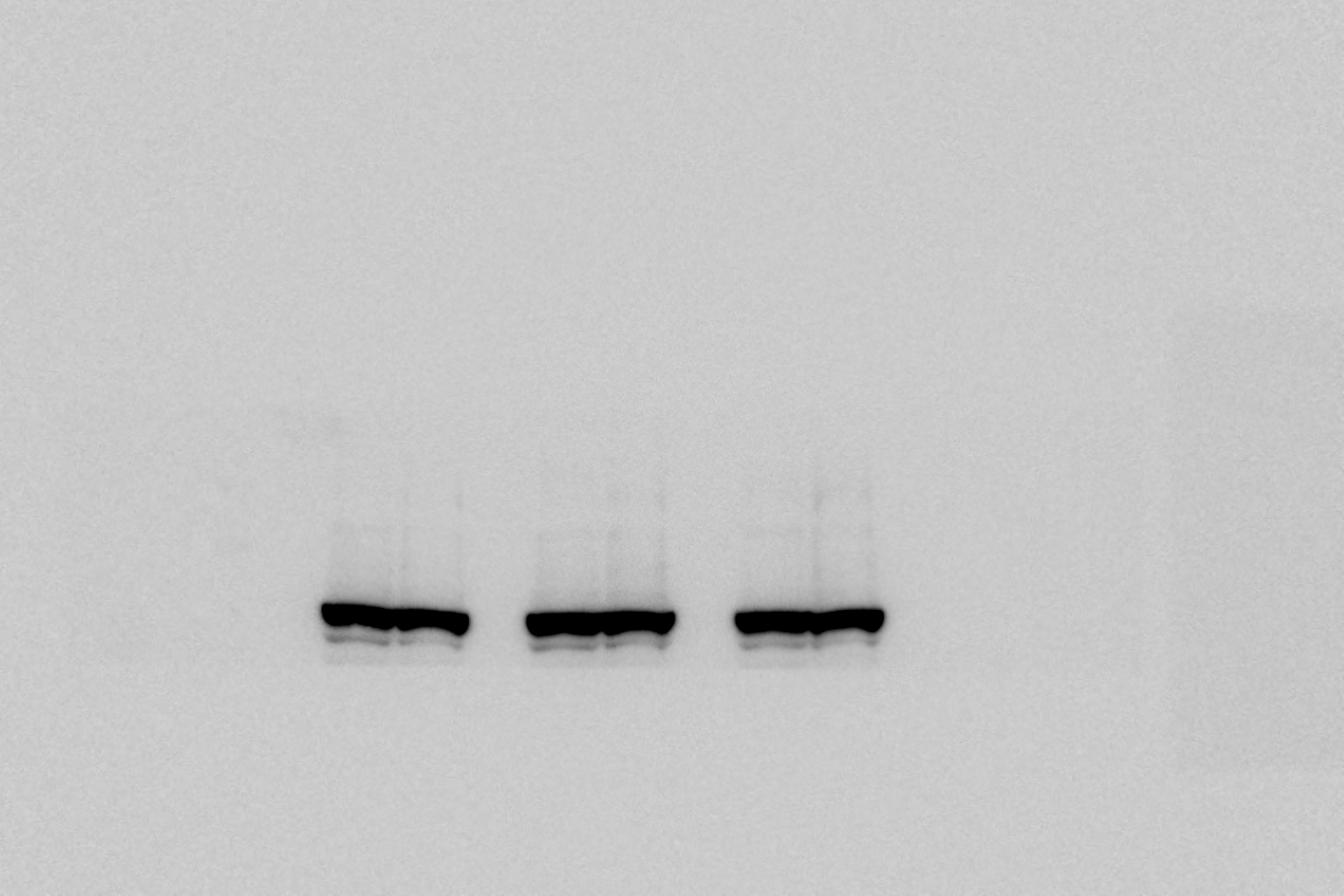

Supplement: Figure 1—source data 1. [file elife-74805-fig1-data1.zip › Fig. 1-source data 1/Fig. 1B(d) Blot SERCA2.tif]

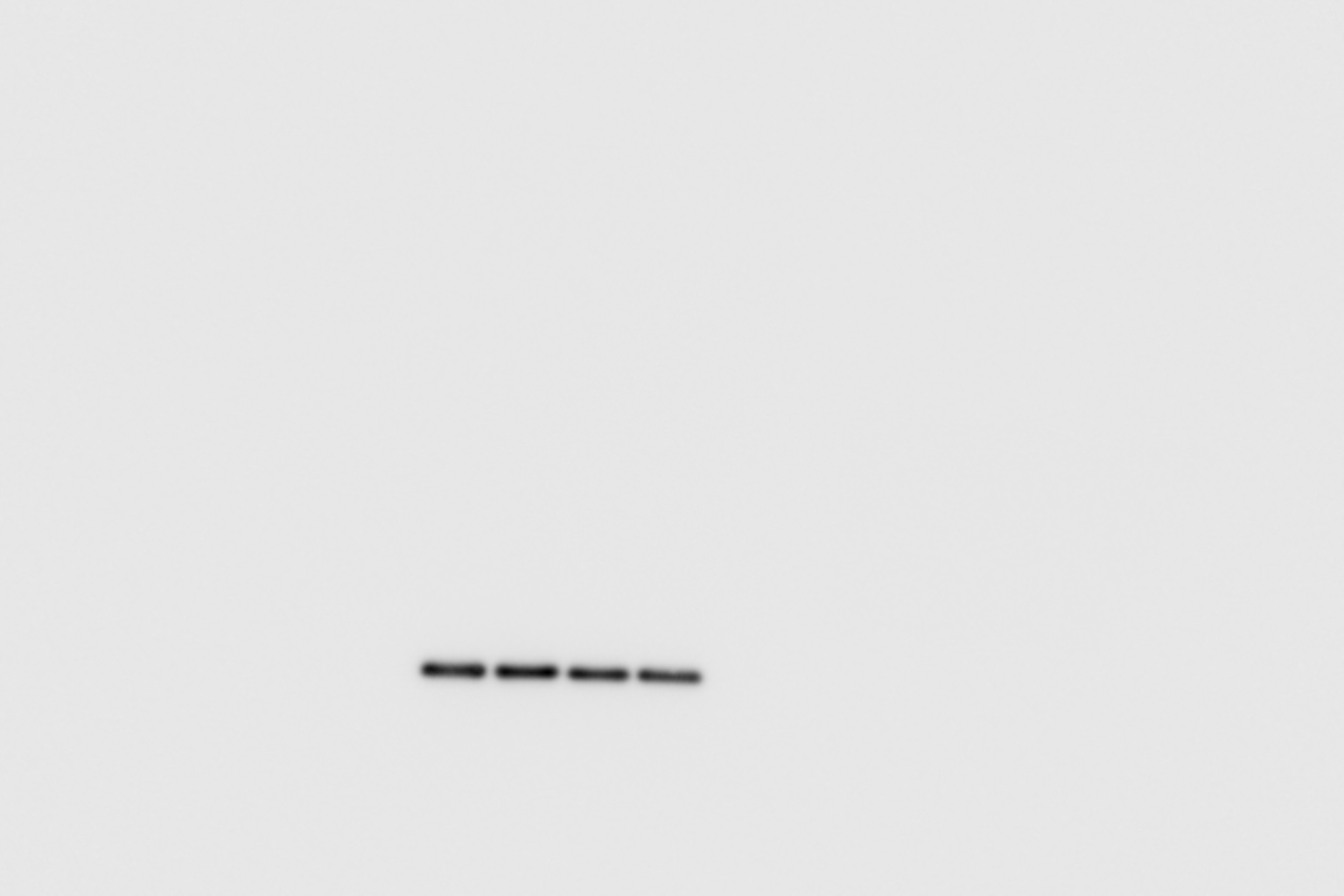

Supplement: Figure 1—source data 1. [file elife-74805-fig1-data1.zip › Fig. 1-source data 1/Fig. 1B(a) Blot GAPDH.tif]

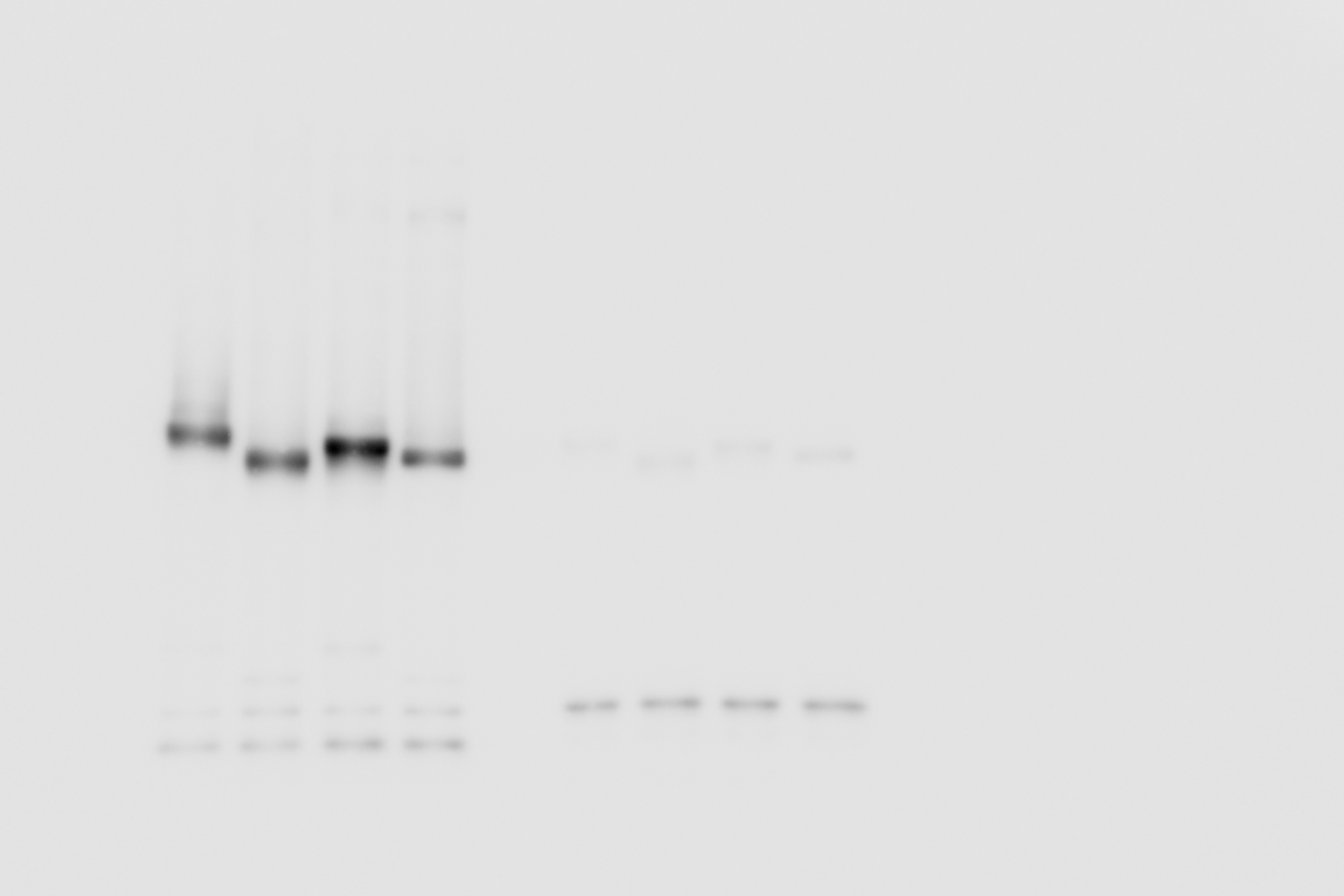

Supplement: Figure 1—source data 1. [file elife-74805-fig1-data1.zip › Fig. 1-source data 1/Fig. 1C Blot Input-Seipin .tif]

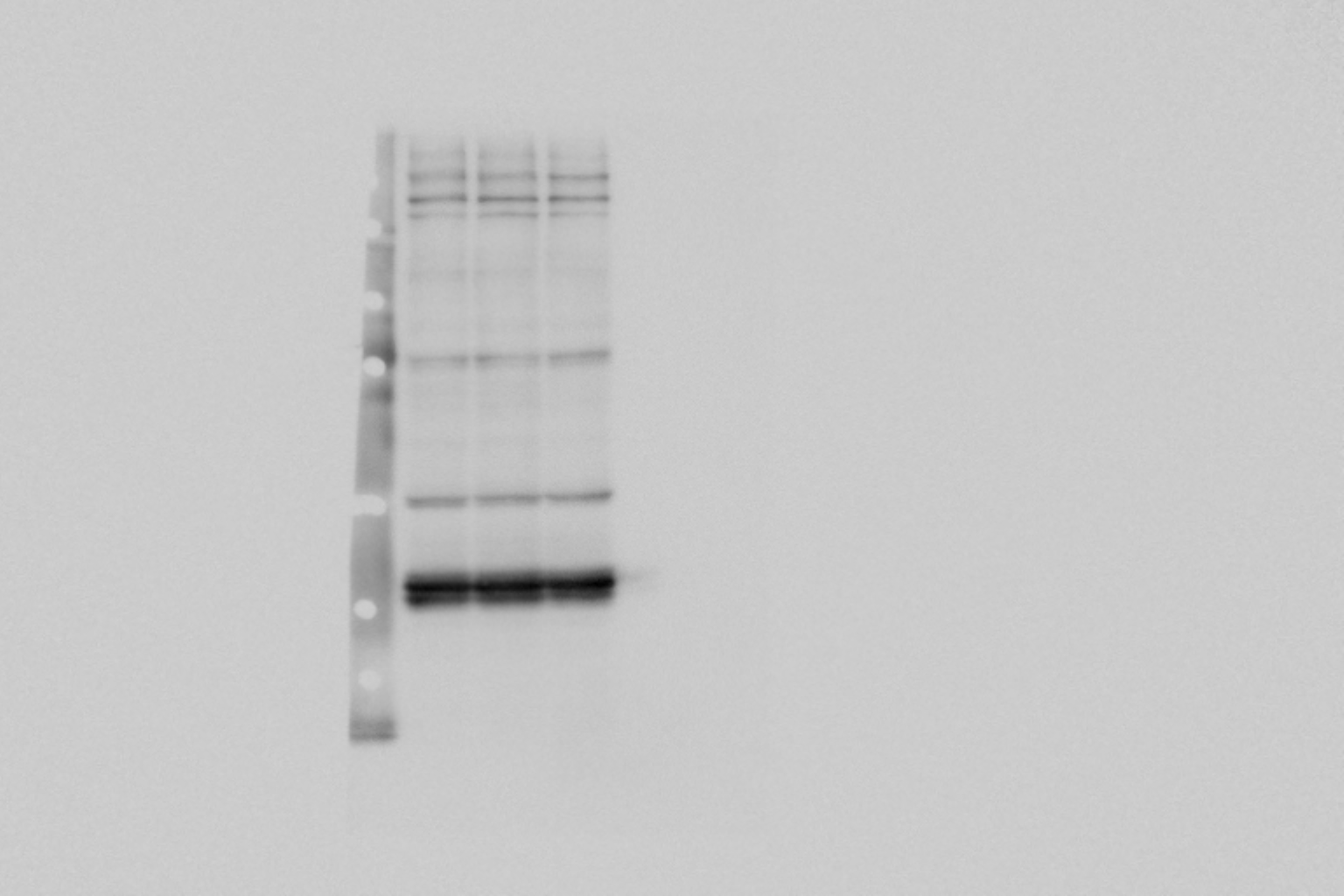

Supplement: Figure 1—source data 1. [file elife-74805-fig1-data1.zip › Fig. 1-source data 1/Fig. 1E Blot G-Cepia1er .tif]

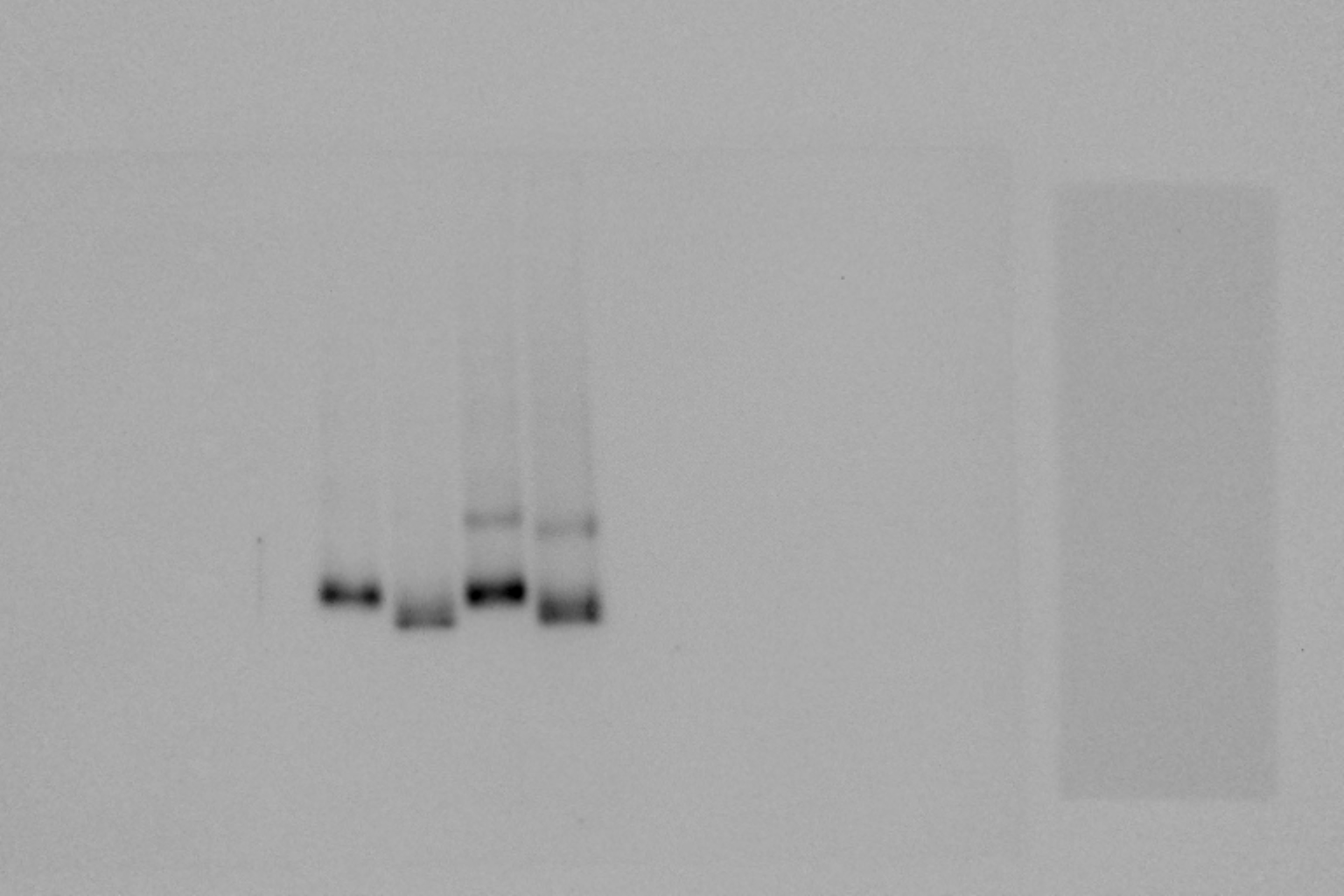

Supplement: Figure 1—source data 1. [file elife-74805-fig1-data1.zip › Fig. 1-source data 1/FIg. 1B(a) Blot Seipin.tif]

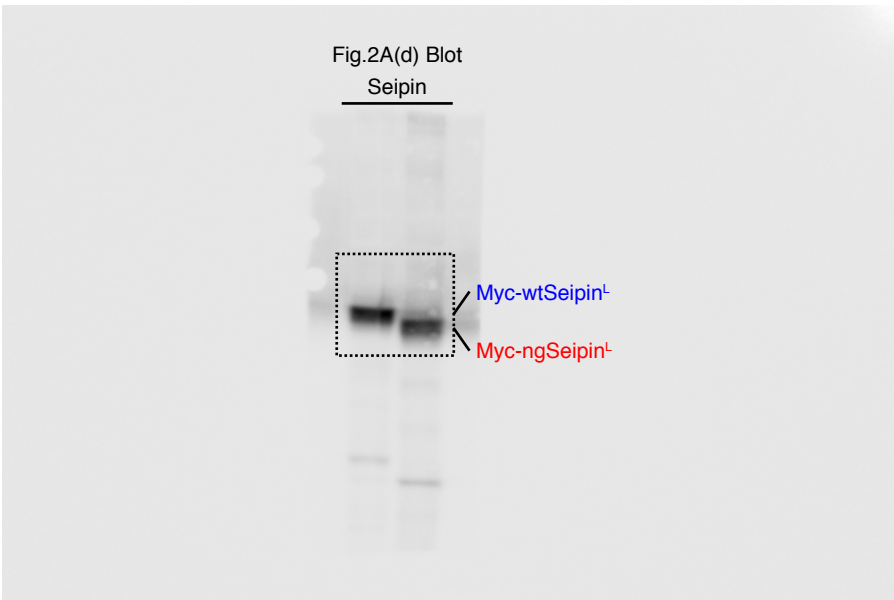

Mouse Anti-myc Direct

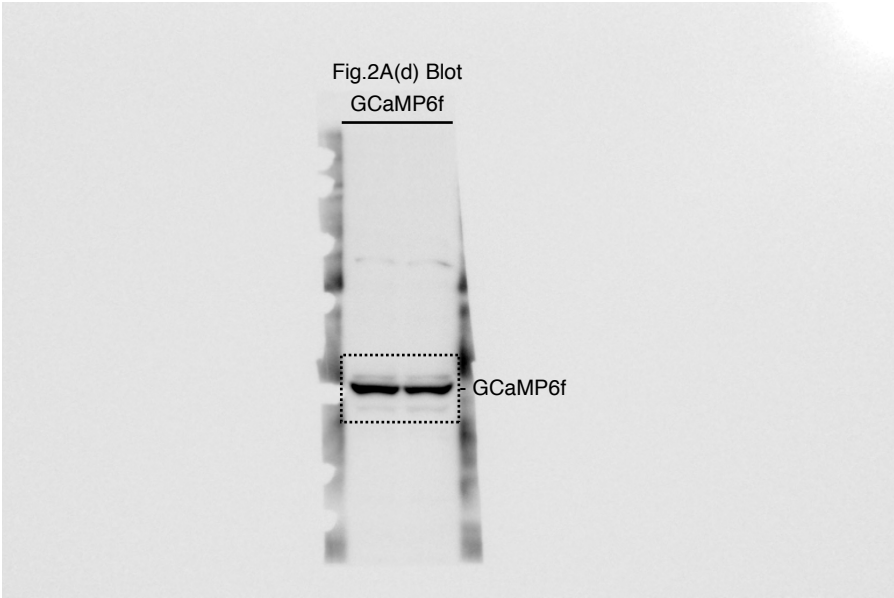

Rabbit Anti-GFP

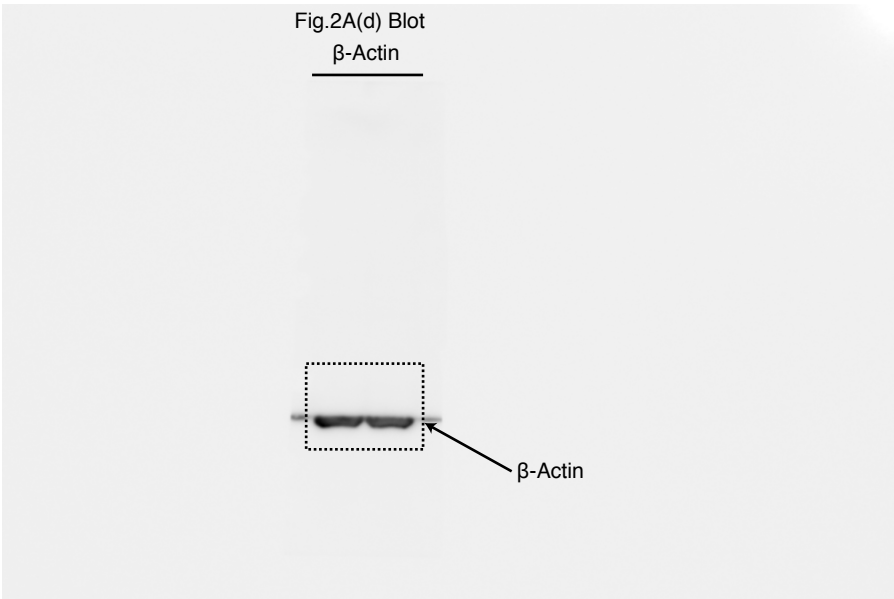

Mouse Anti- $\beta$ -Actin

Supplement: Figure 2—source data 1. [file elife-74805-fig2-data1.zip › Fig. 2-source data 1/Fig. 2-source data.pdf]

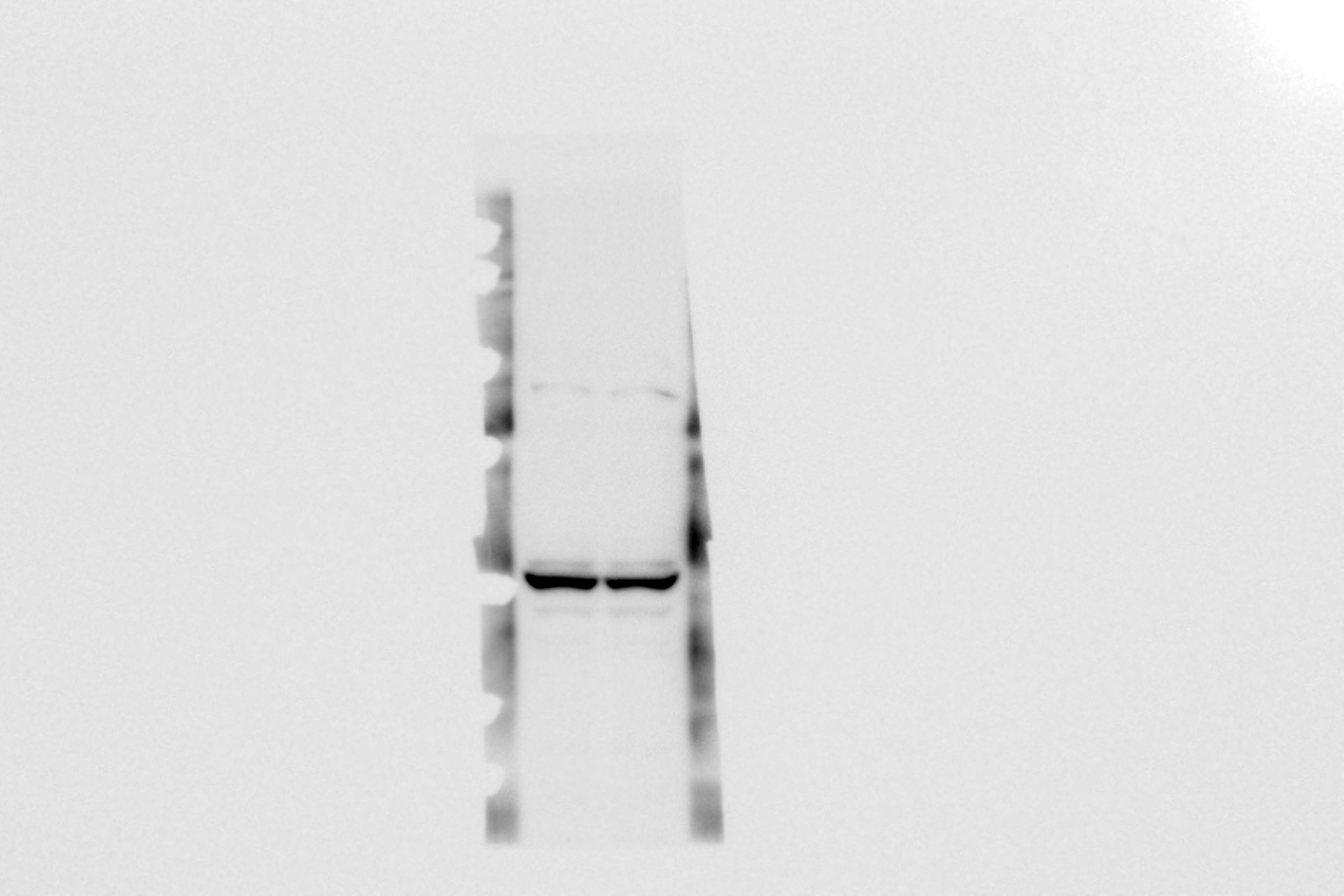

Supplement: Figure 2—source data 1. [file elife-74805-fig2-data1.zip › Fig. 2-source data 1/Fig. 2A(d) Blot GCaMP6f.tif]

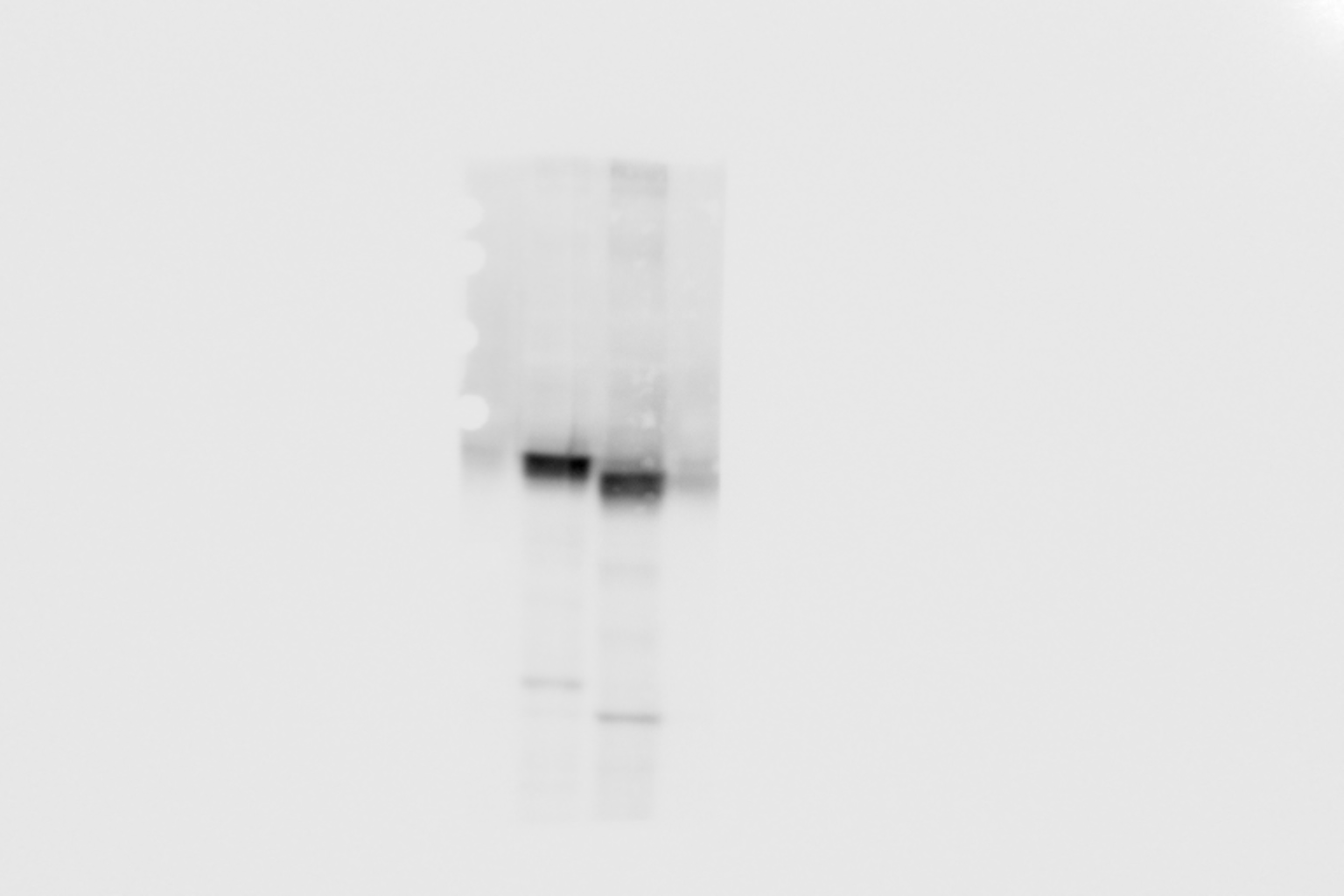

Supplement: Figure 2—source data 1. [file elife-74805-fig2-data1.zip › Fig. 2-source data 1/Fig. 2A(d) Blot Seipin.tif]

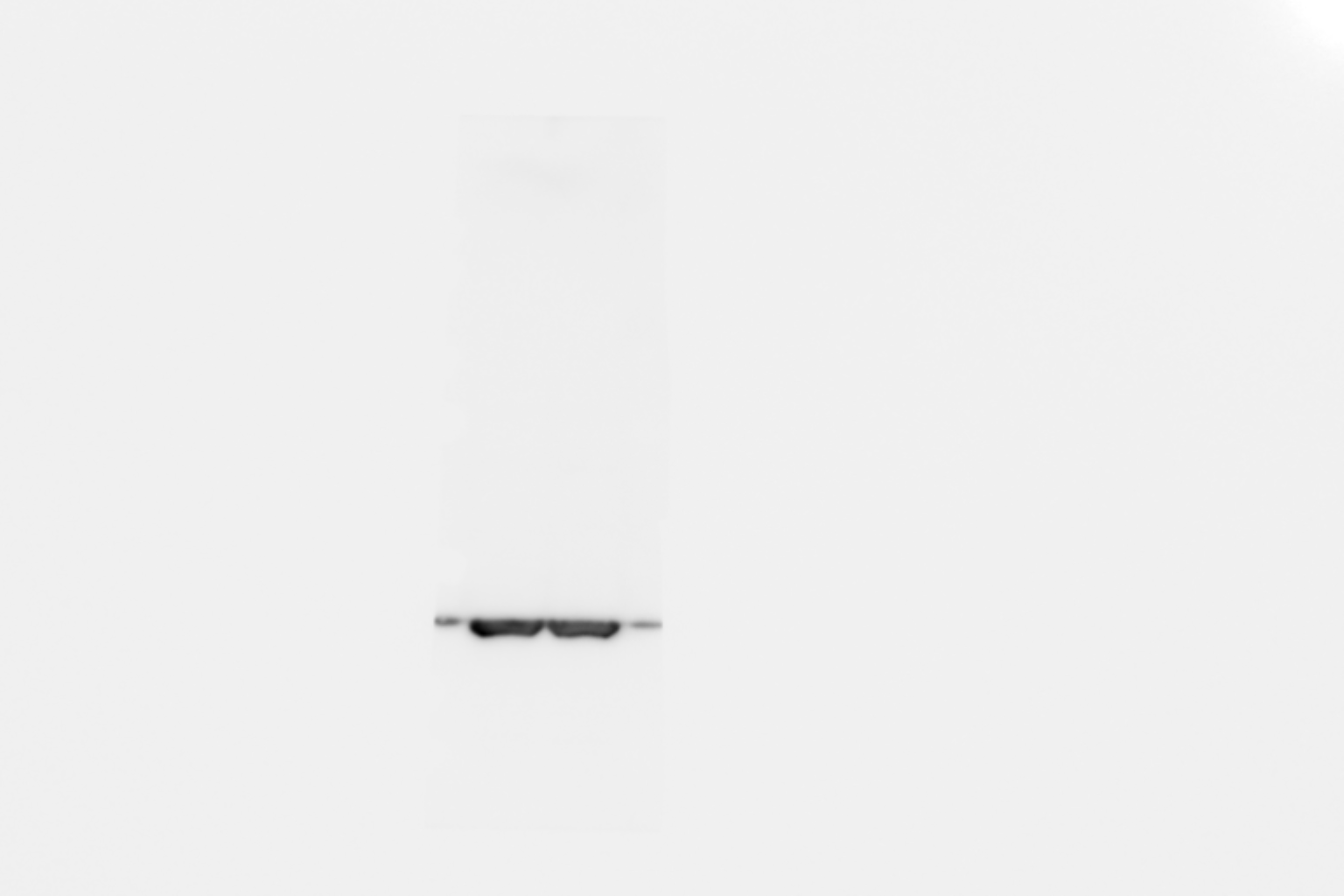

Supplement: Figure 2—source data 1. [file elife-74805-fig2-data1.zip › Fig. 2-source data 1/Fig. 2A(d) Blot b-Actin .tif]

Fig.2-S1-source data

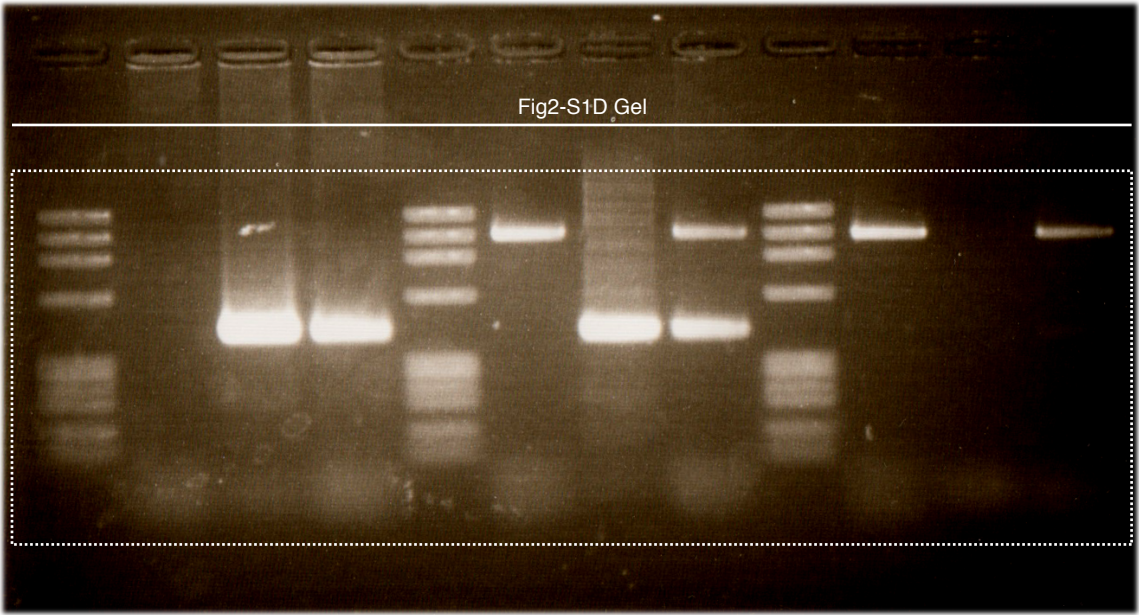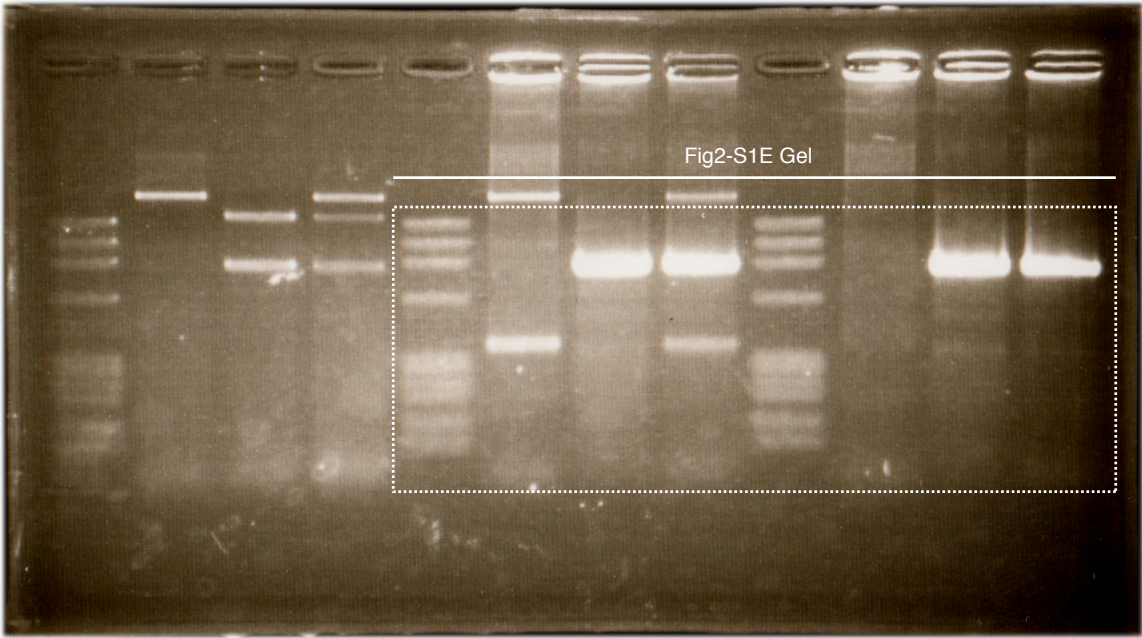

Supplement: Figure 2—figure supplement 1—source data 1. [file elife-74805-fig2-figsupp1-data1.zip › Fig. 2-S1-source data 1/Fig. 2-S1-source data.pdf]

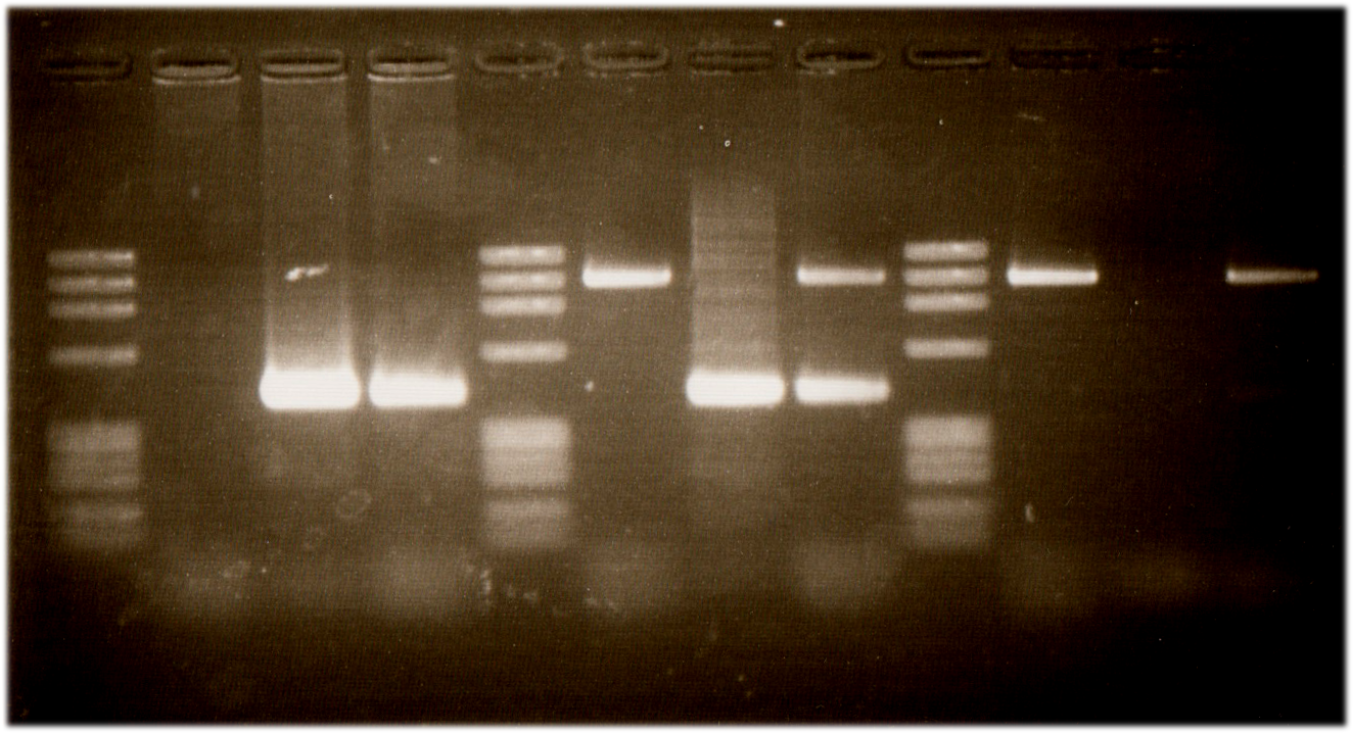

Supplement: Figure 2—figure supplement 1—source data 1. [file elife-74805-fig2-figsupp1-data1.zip › Fig. 2-S1-source data 1/Fig. 2-S1D Gel.tif]

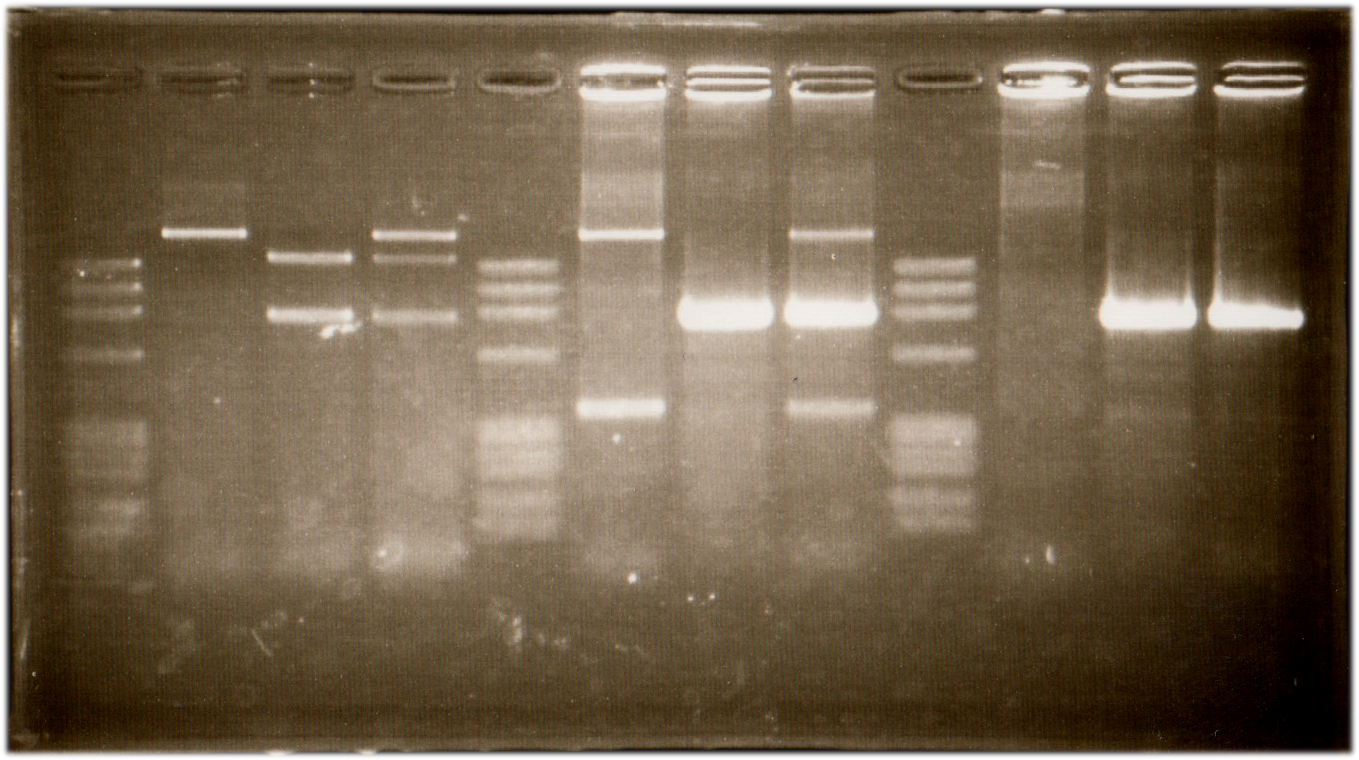

Supplement: Figure 2—figure supplement 1—source data 1. [file elife-74805-fig2-figsupp1-data1.zip › Fig. 2-S1-source data 1/Fig. 2-S1E Gel.tif]

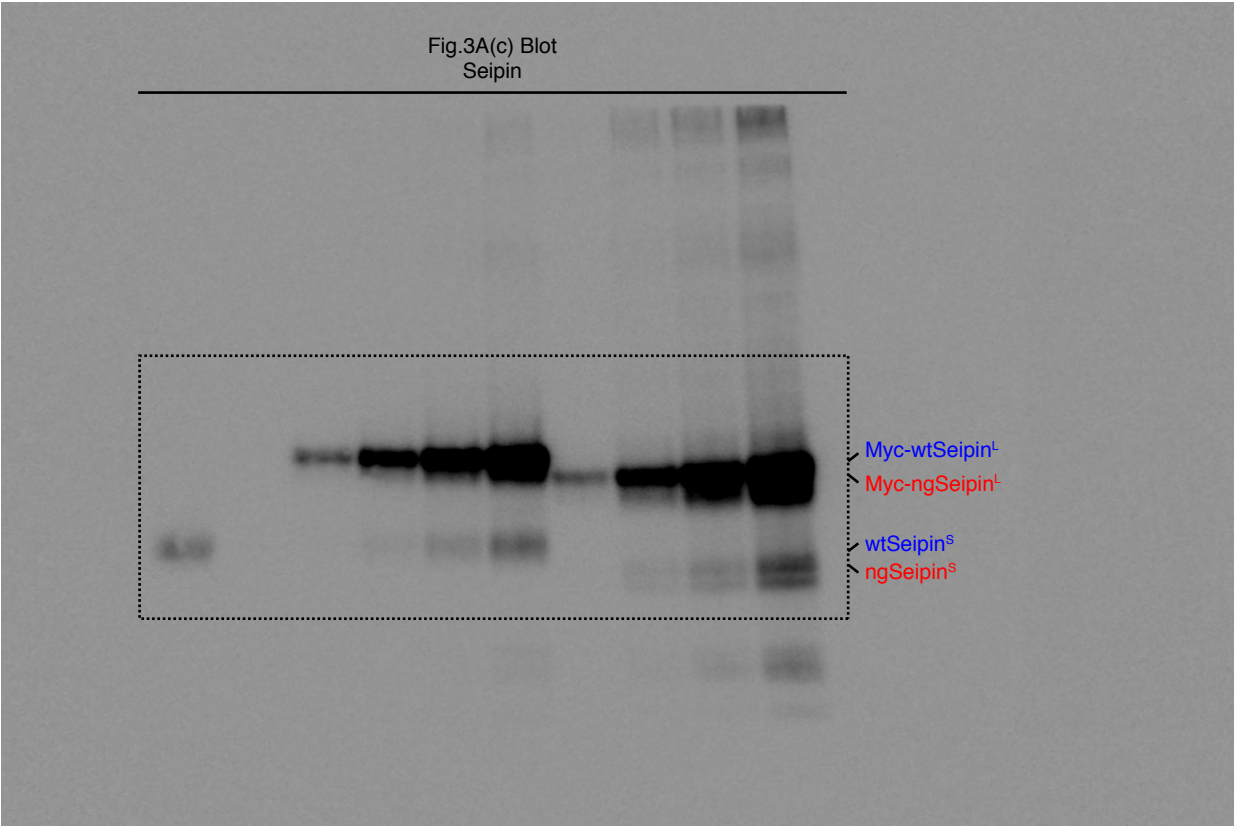

Rabbit Anti-Seipin

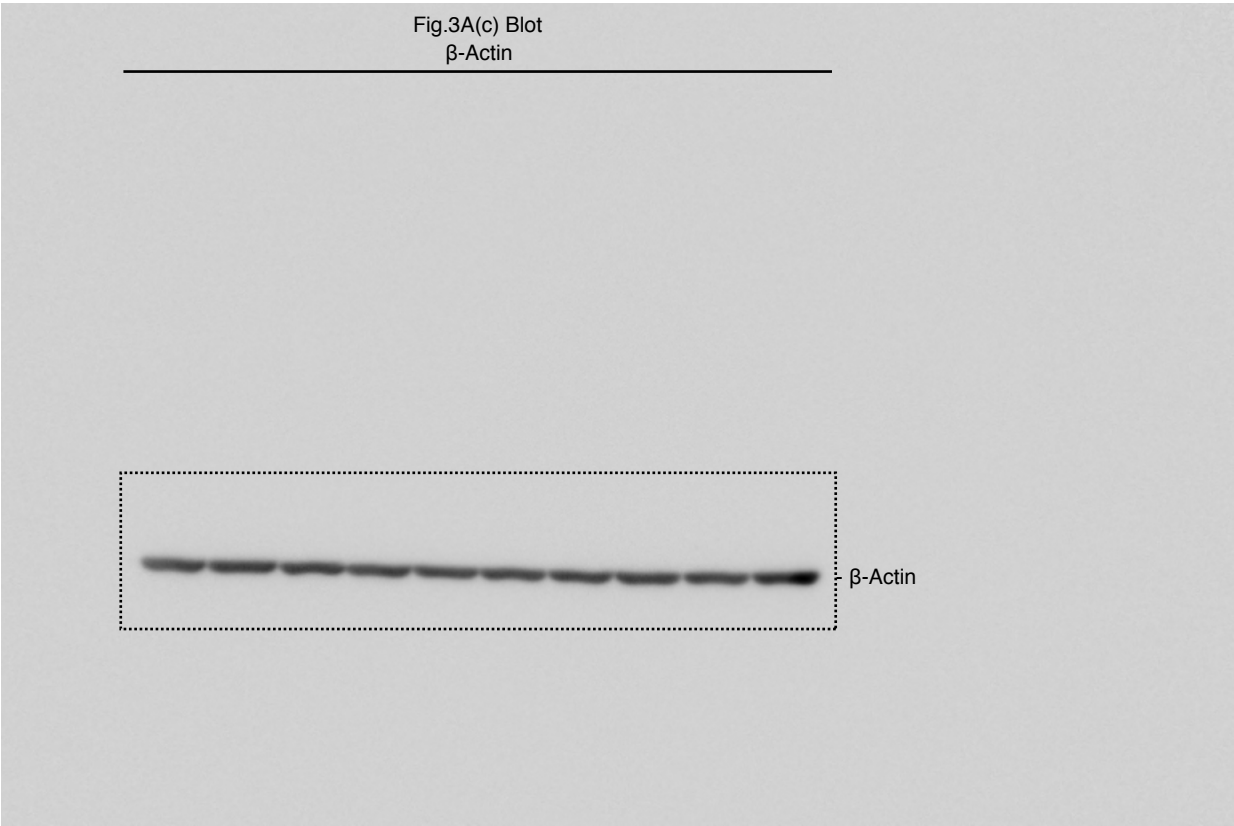

Mouse Anti- $\beta$ -Actin

Supplement: Figure 3—source data 1. [file elife-74805-fig3-data1.zip › Fig. 3-source data 1/Fig. 3-source data.pdf]

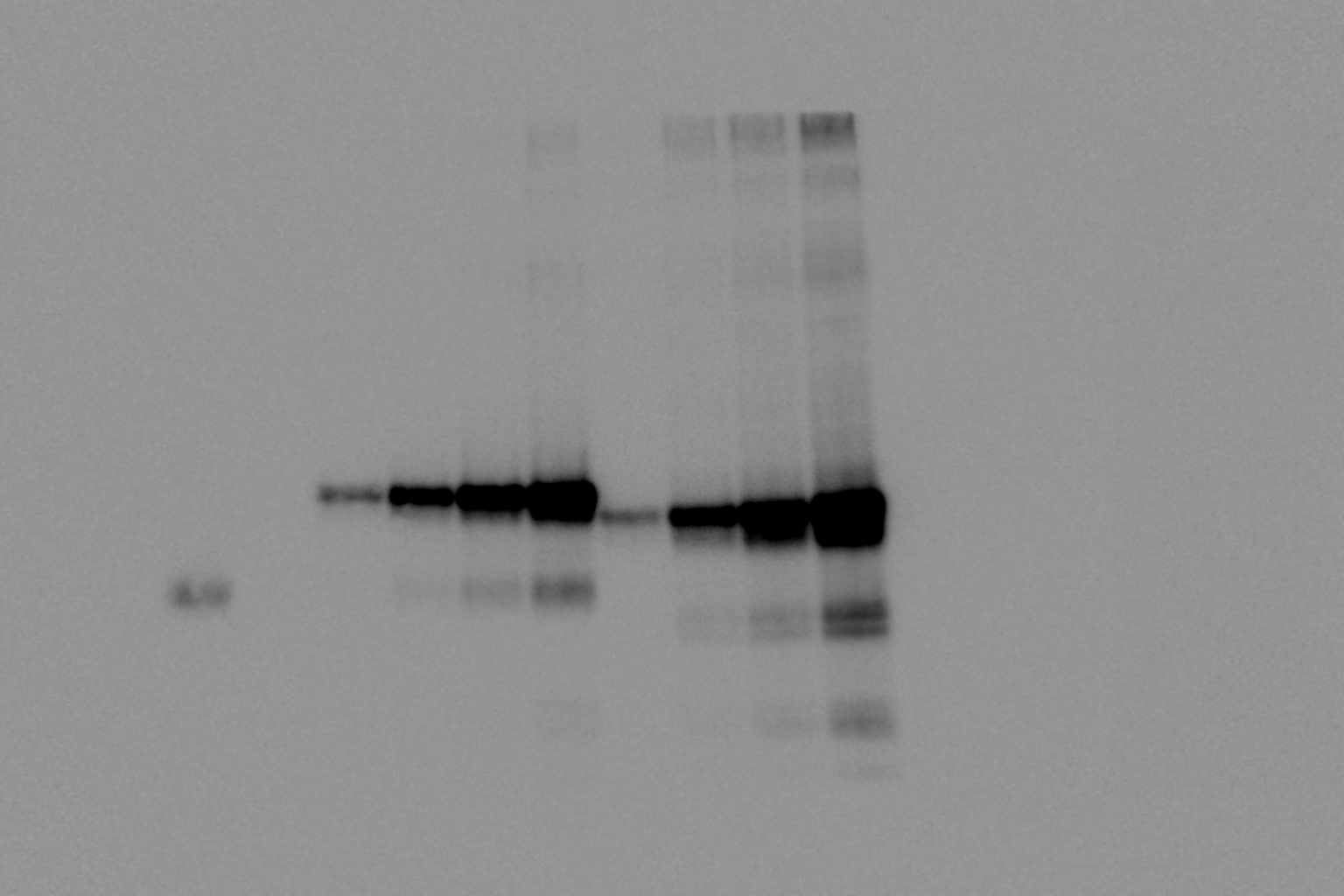

Supplement: Figure 3—source data 1. [file elife-74805-fig3-data1.zip › Fig. 3-source data 1/Fig. 3A(c) Blot Seipin.tif]

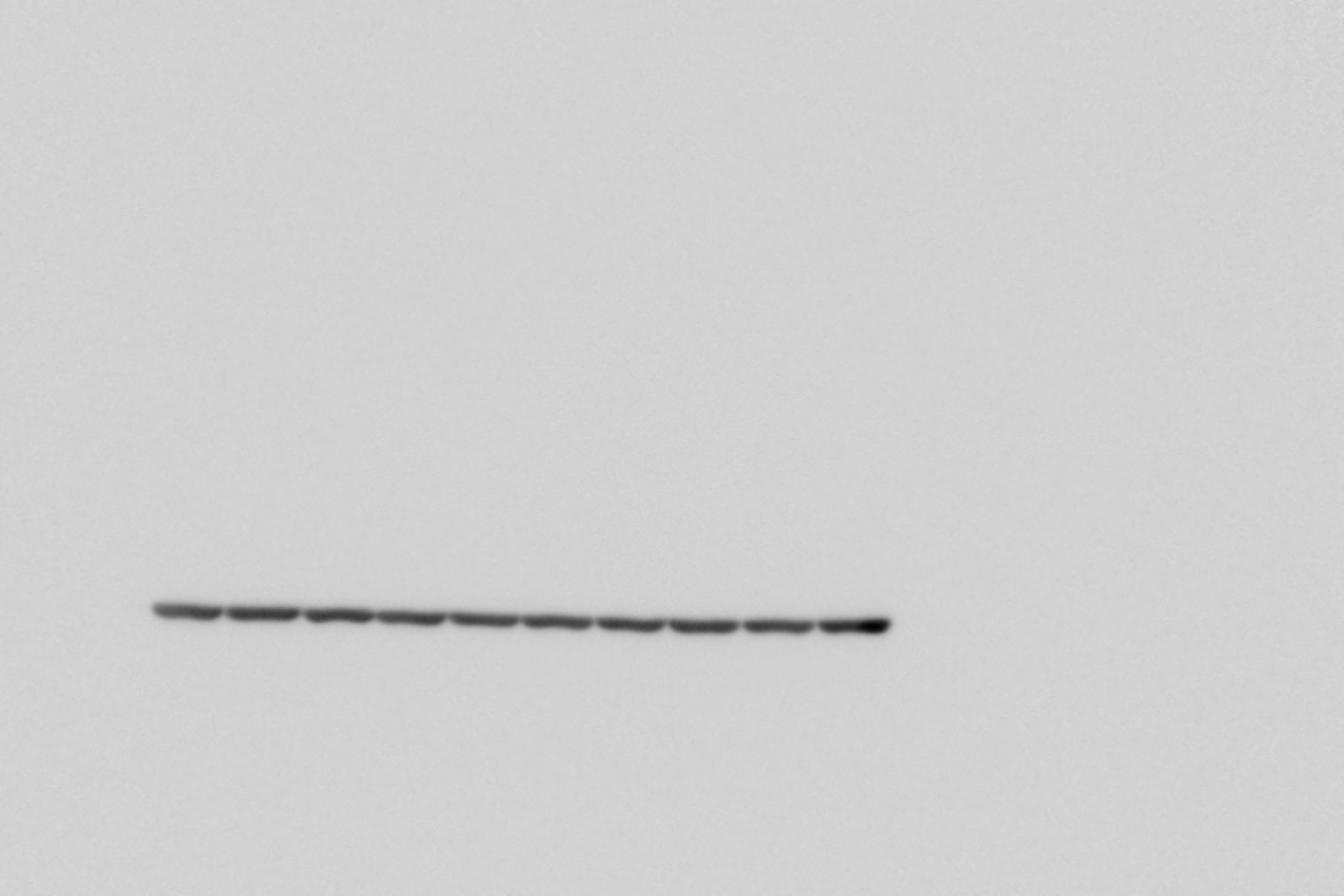

Supplement: Figure 3—source data 1. [file elife-74805-fig3-data1.zip › Fig. 3-source data 1/Fig. 3A(c) Blot b-Actin.tif]

**Fig.4-source data**

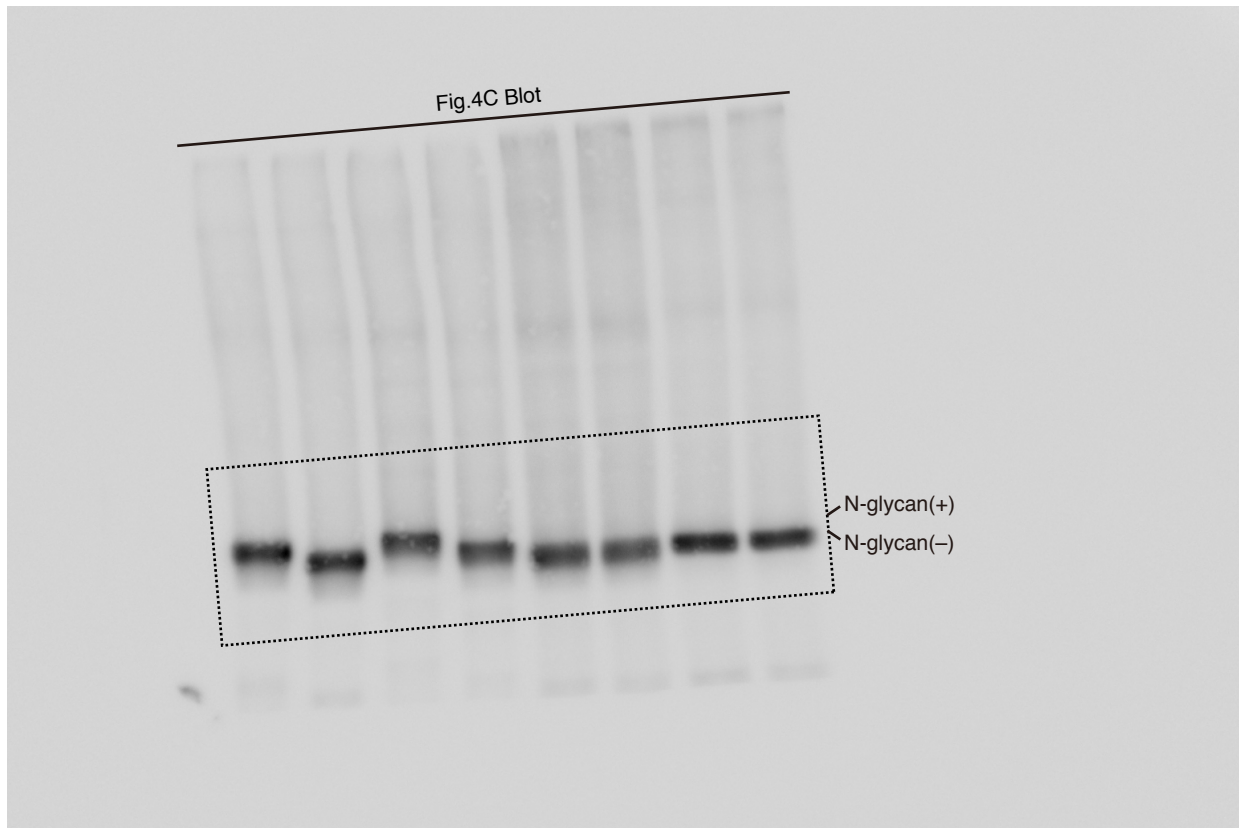

Mouse Anti-myc Direct

Supplement: Figure 4—source data 1. [file elife-74805-fig4-data1.zip › Fig. 4-source data 1/Fig. 4-source data.pdf]

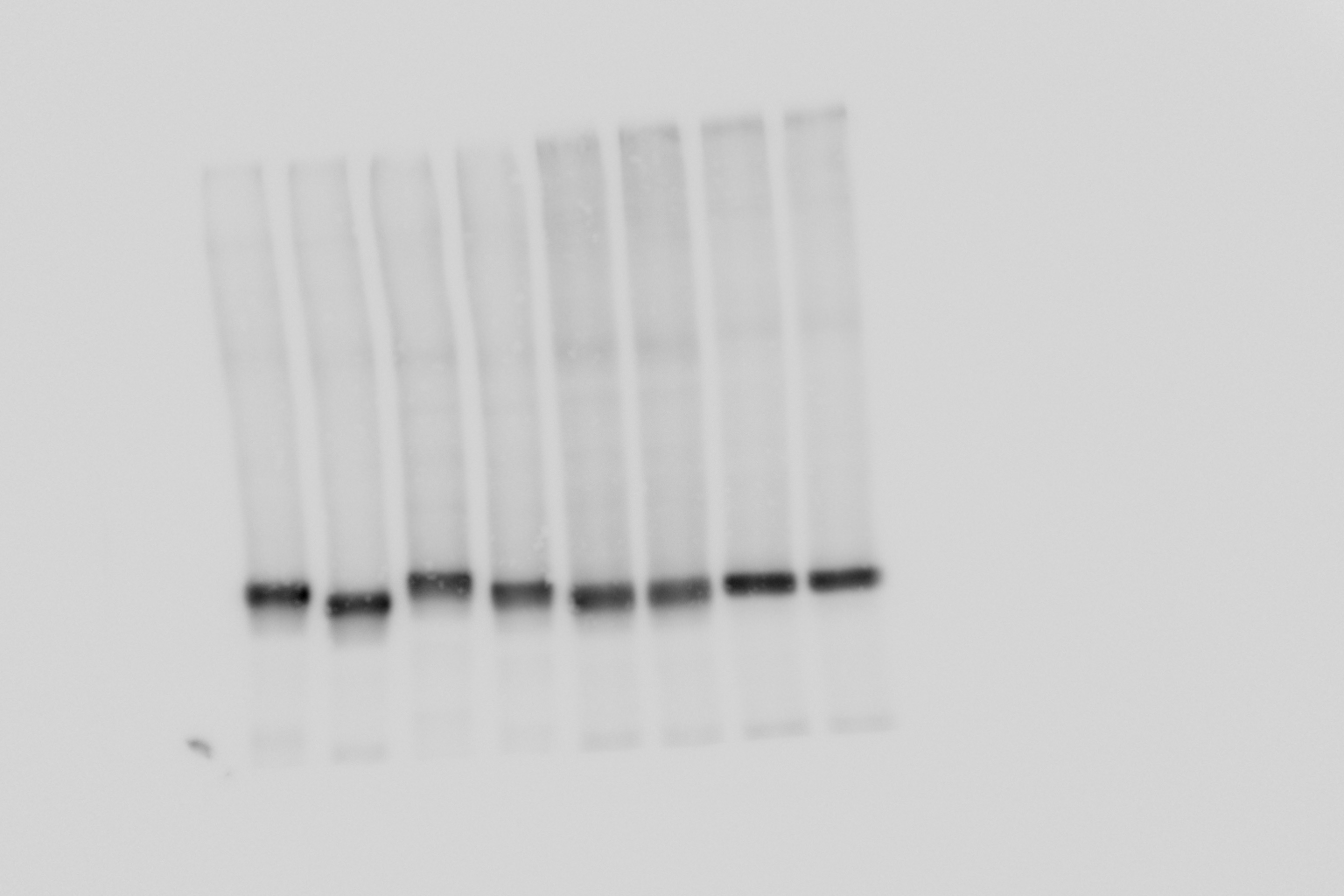

Supplement: Figure 4—source data 1. [file elife-74805-fig4-data1.zip › Fig. 4-source data 1/Fig. 4C Blot.tif]

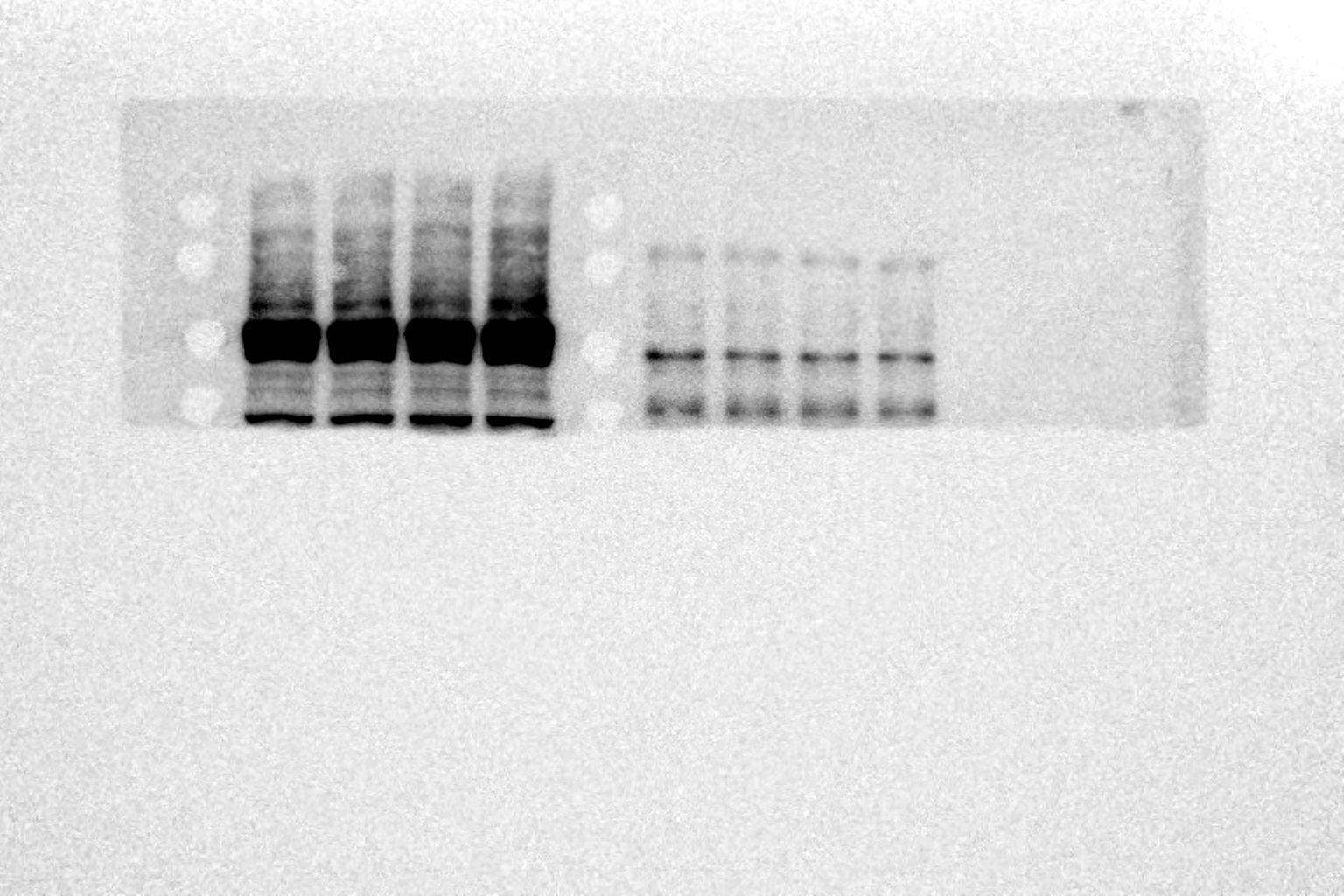

Supplement: Figure 5—source data 1. [file elife-74805-fig5-data1.zip › Fig. 5-source data 1/Fig. 5A Blot IP-SERCA2.tif]

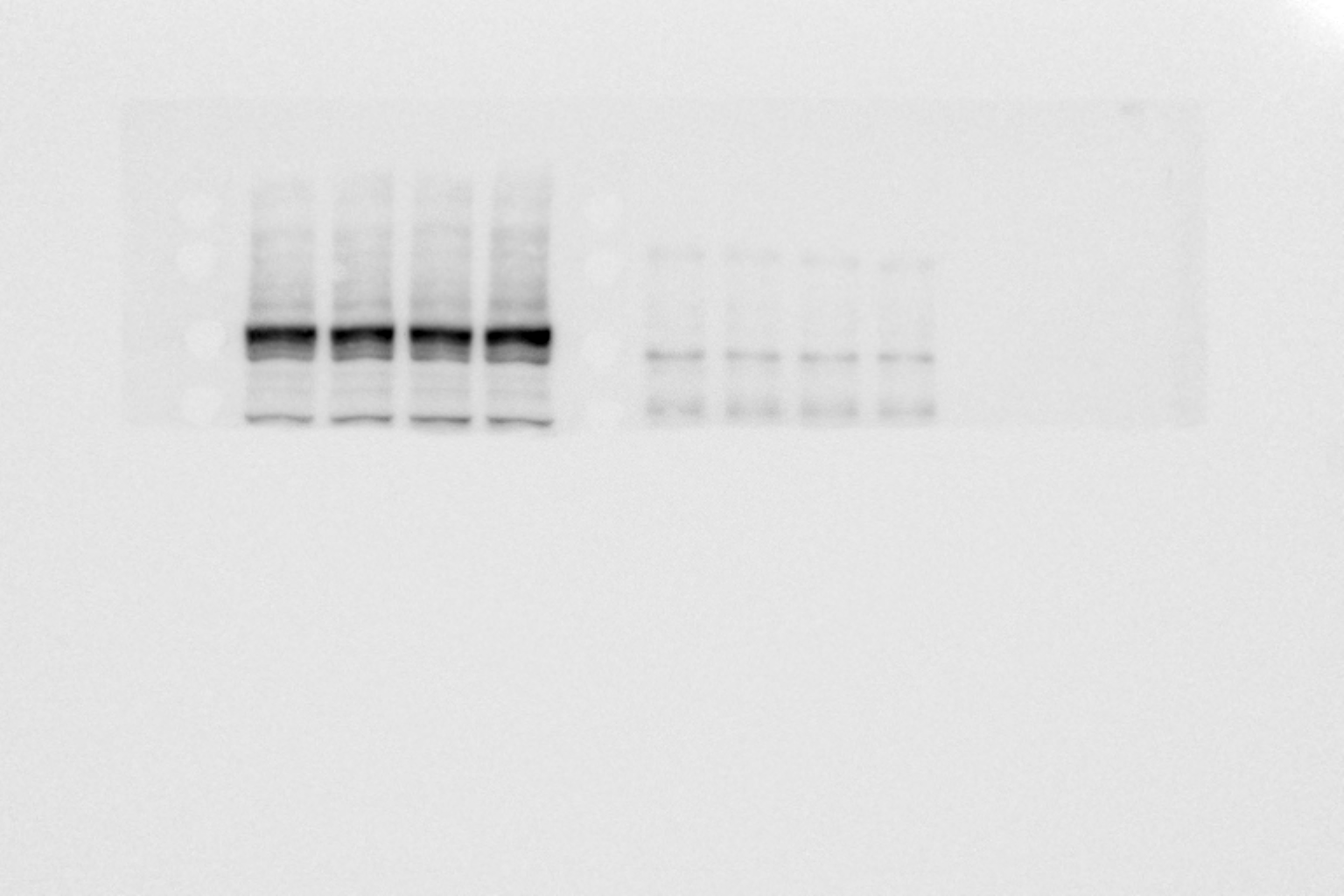

Supplement: Figure 5—source data 1. [file elife-74805-fig5-data1.zip › Fig. 5-source data 1/Fig. 5A Blot Input-SERCA2.tif]

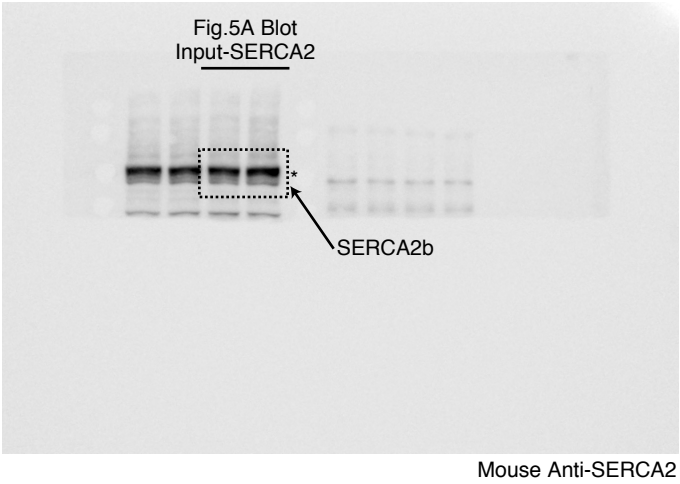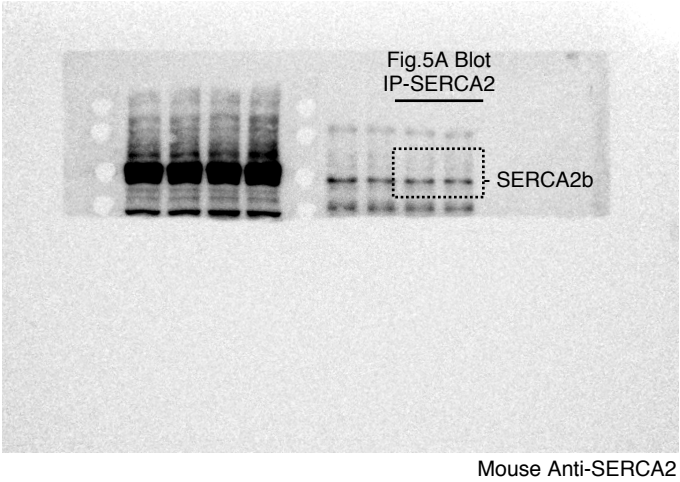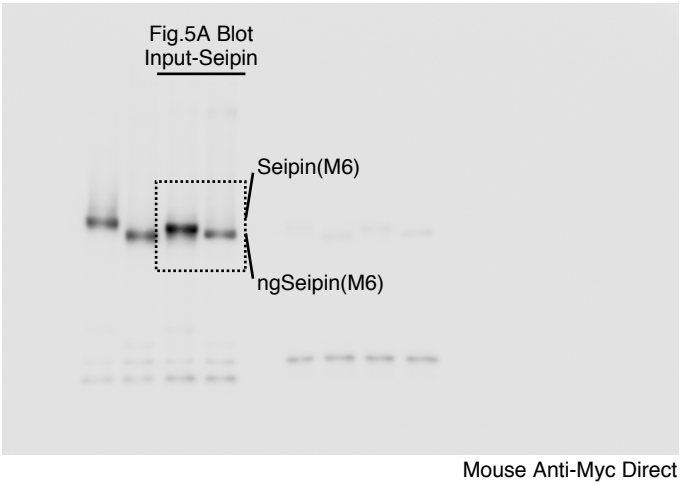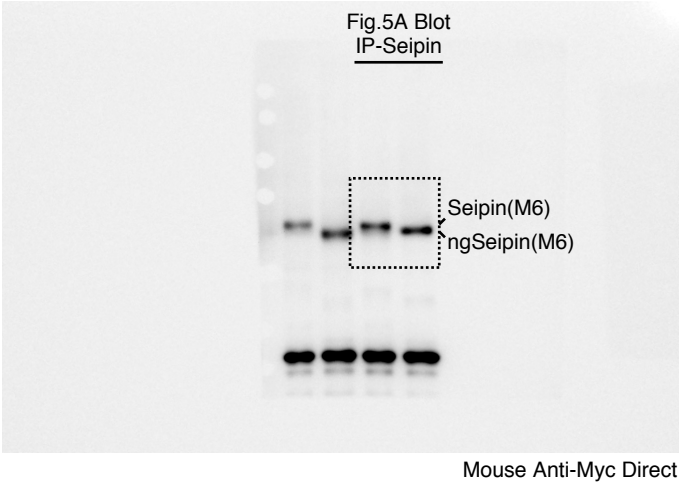

Supplement: Figure 5—source data 1. [file elife-74805-fig5-data1.zip › Fig. 5-source data 1/Fig.5-source data.pdf]

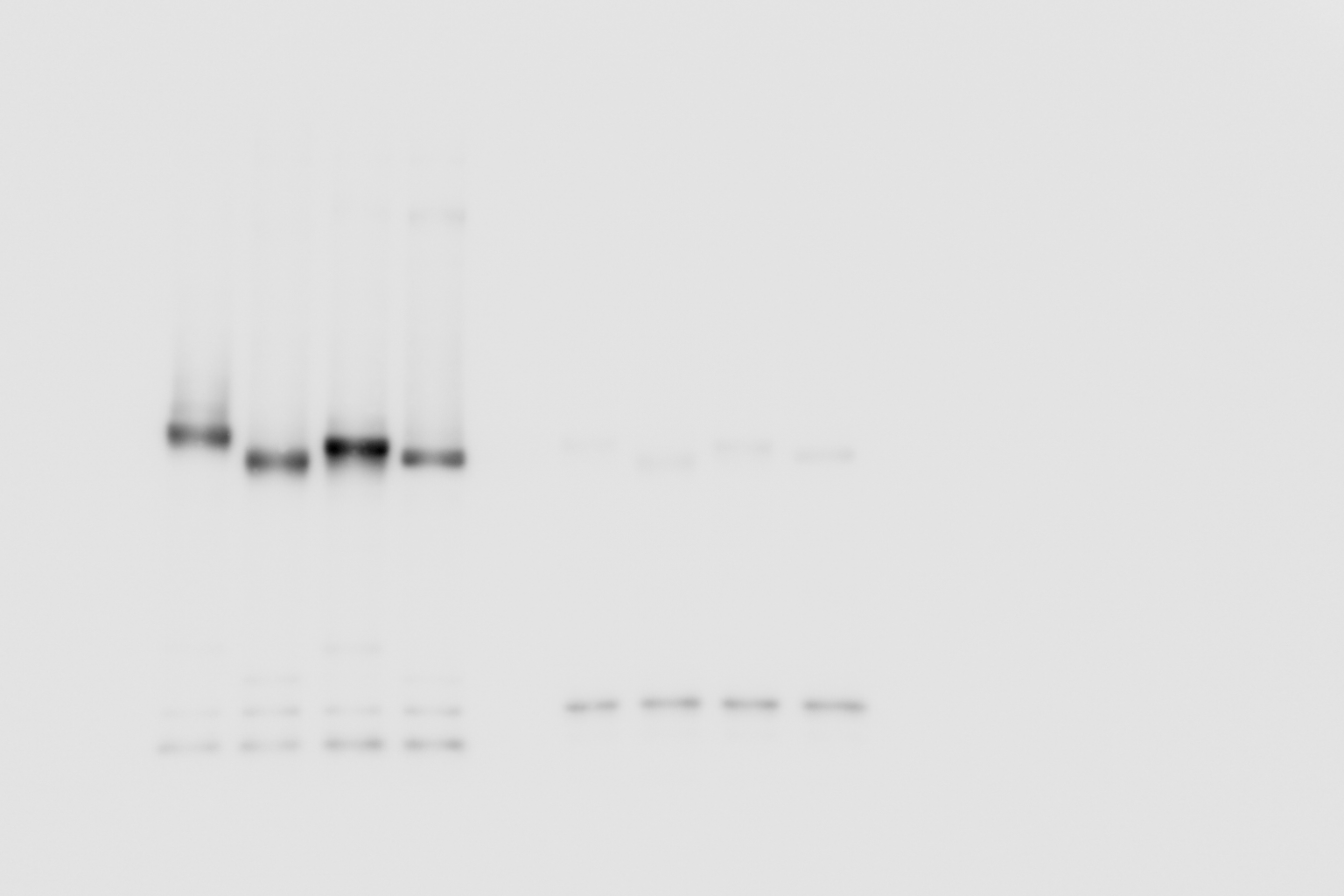

Supplement: Figure 5—source data 1. [file elife-74805-fig5-data1.zip › Fig. 5-source data 1/Fig. 5A Blot Input-Seipin.tif]

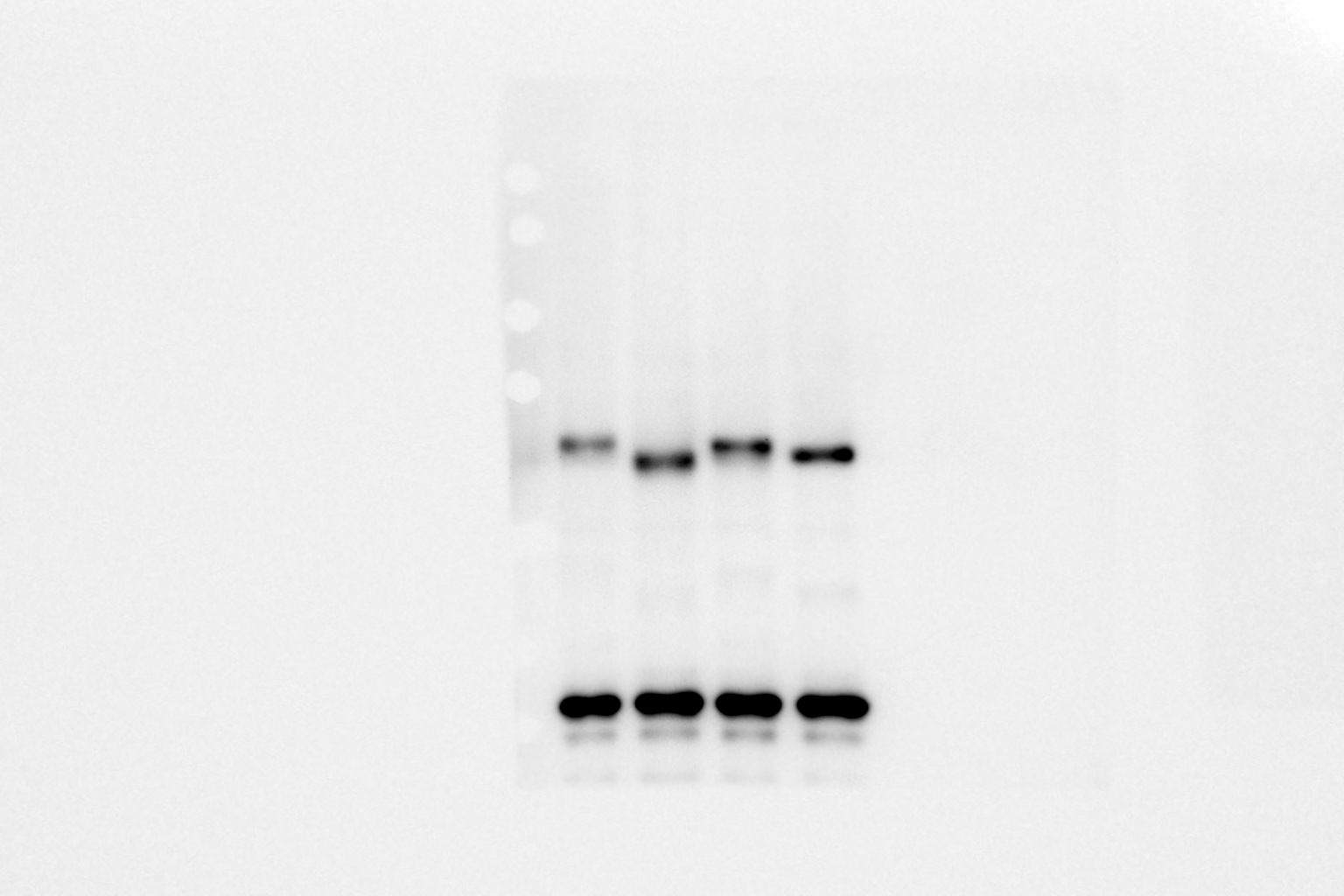

Supplement: Figure 5—source data 1. [file elife-74805-fig5-data1.zip › Fig. 5-source data 1/Fig. 5A Blot IP-Seipin.tif]

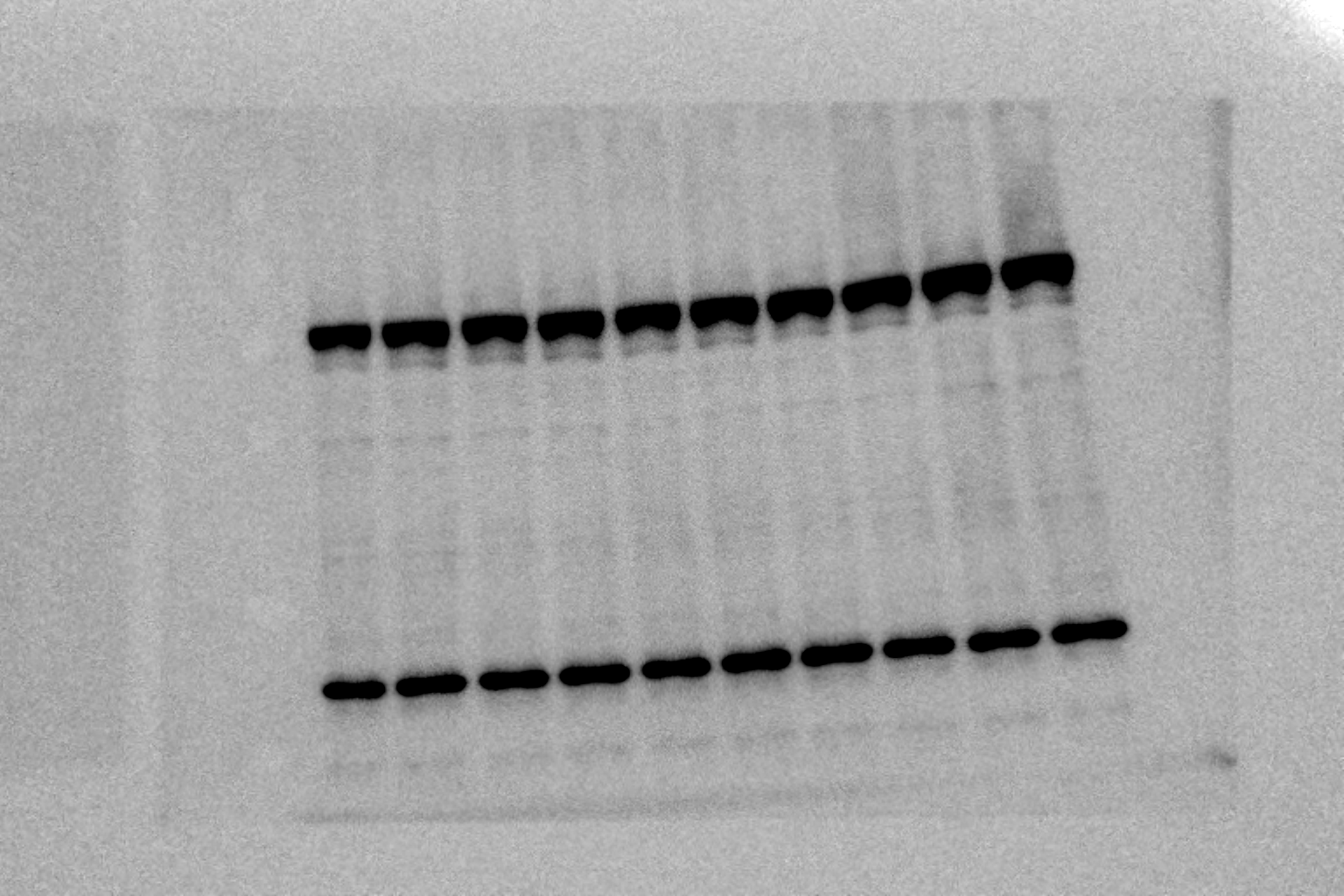

Supplement: Figure 6—source data 1. [file elife-74805-fig6-data1.zip › Fig. 6-source data 1/Fig. 6C Blot Input-SERCA2 .tif]

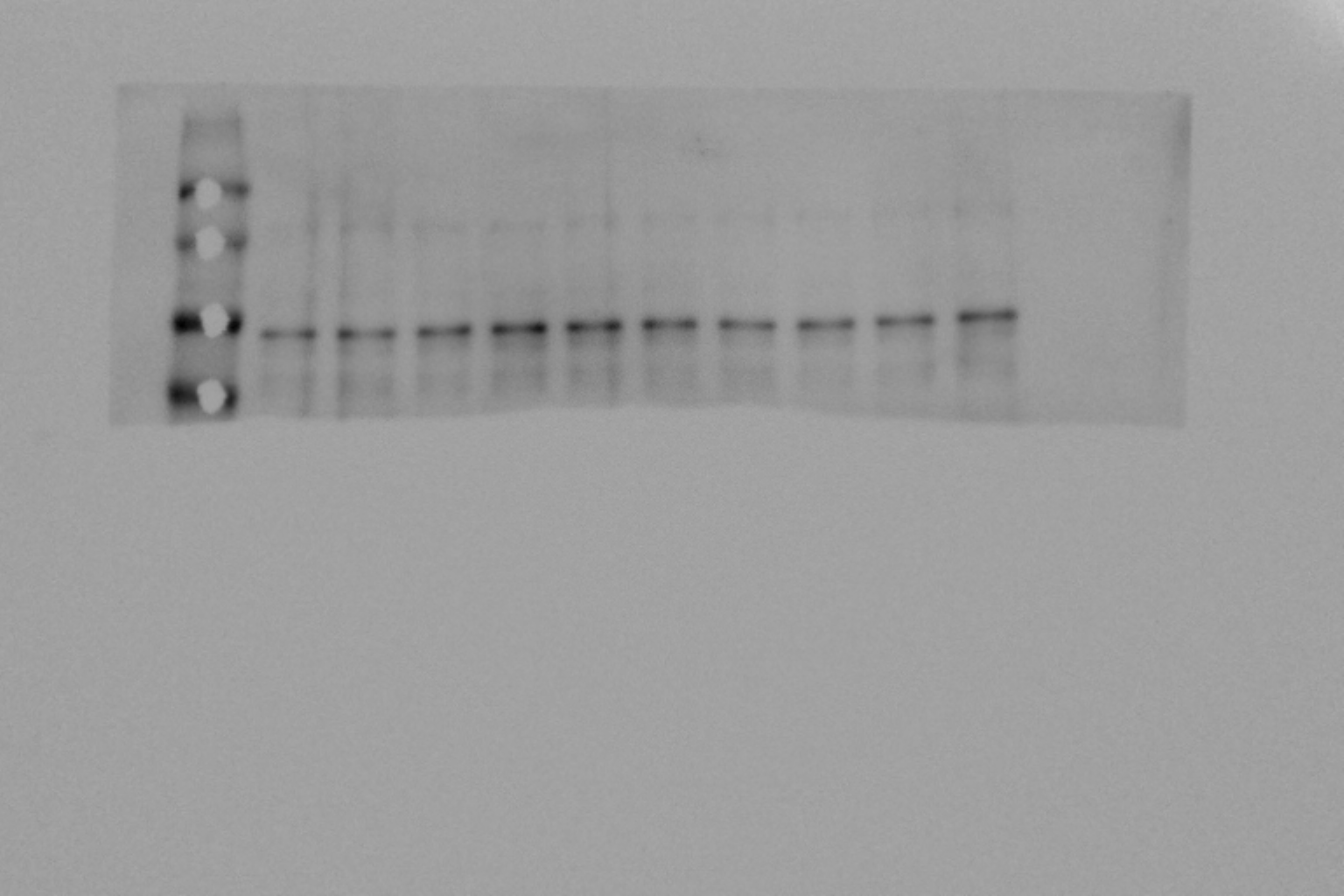

Supplement: Figure 6—source data 1. [file elife-74805-fig6-data1.zip › Fig. 6-source data 1/Fig. 6C Blot IP-SERCA2.tif]

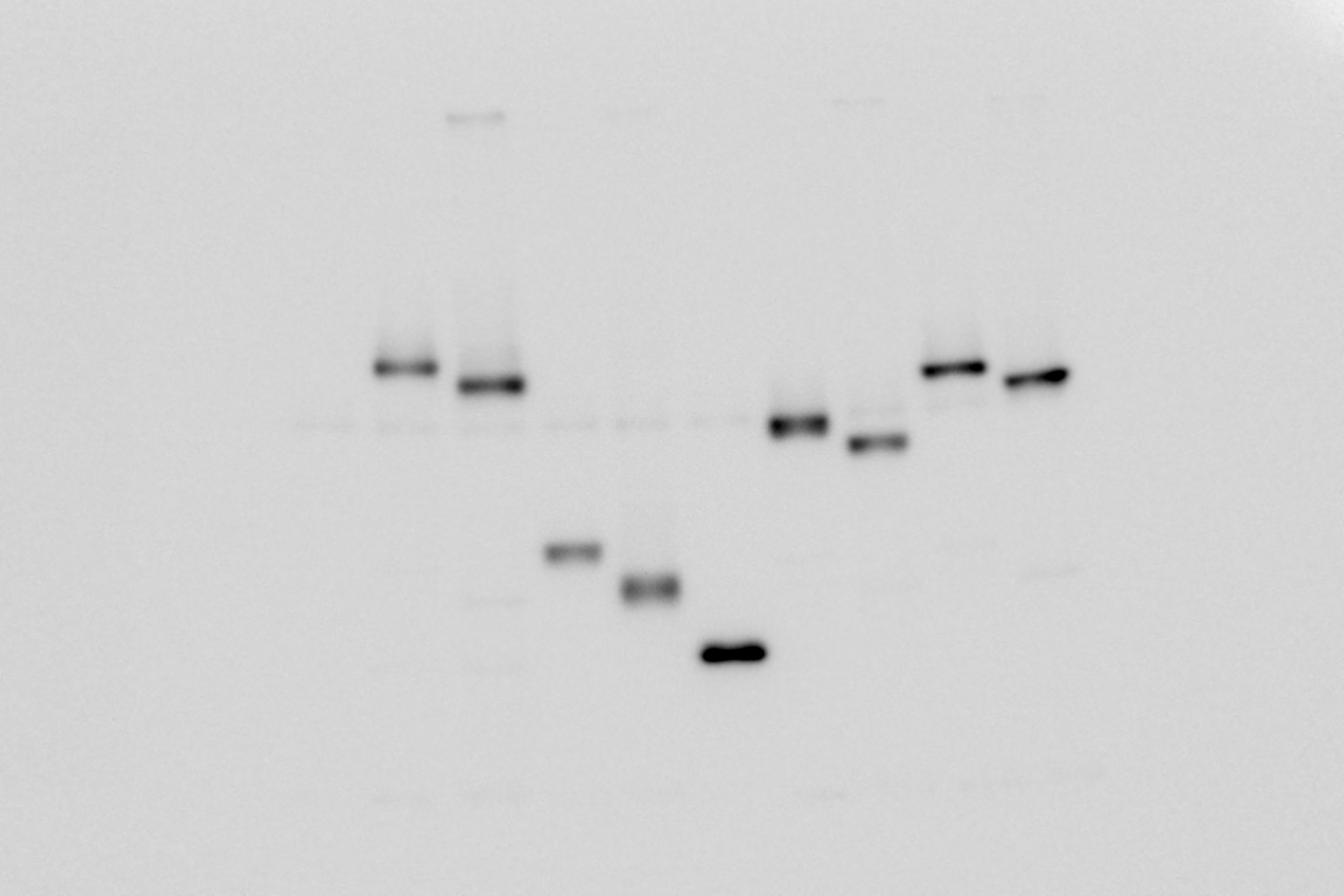

Supplement: Figure 6—source data 1. [file elife-74805-fig6-data1.zip › Fig. 6-source data 1/Fig. 6C Blot Input-Seipin .tif]

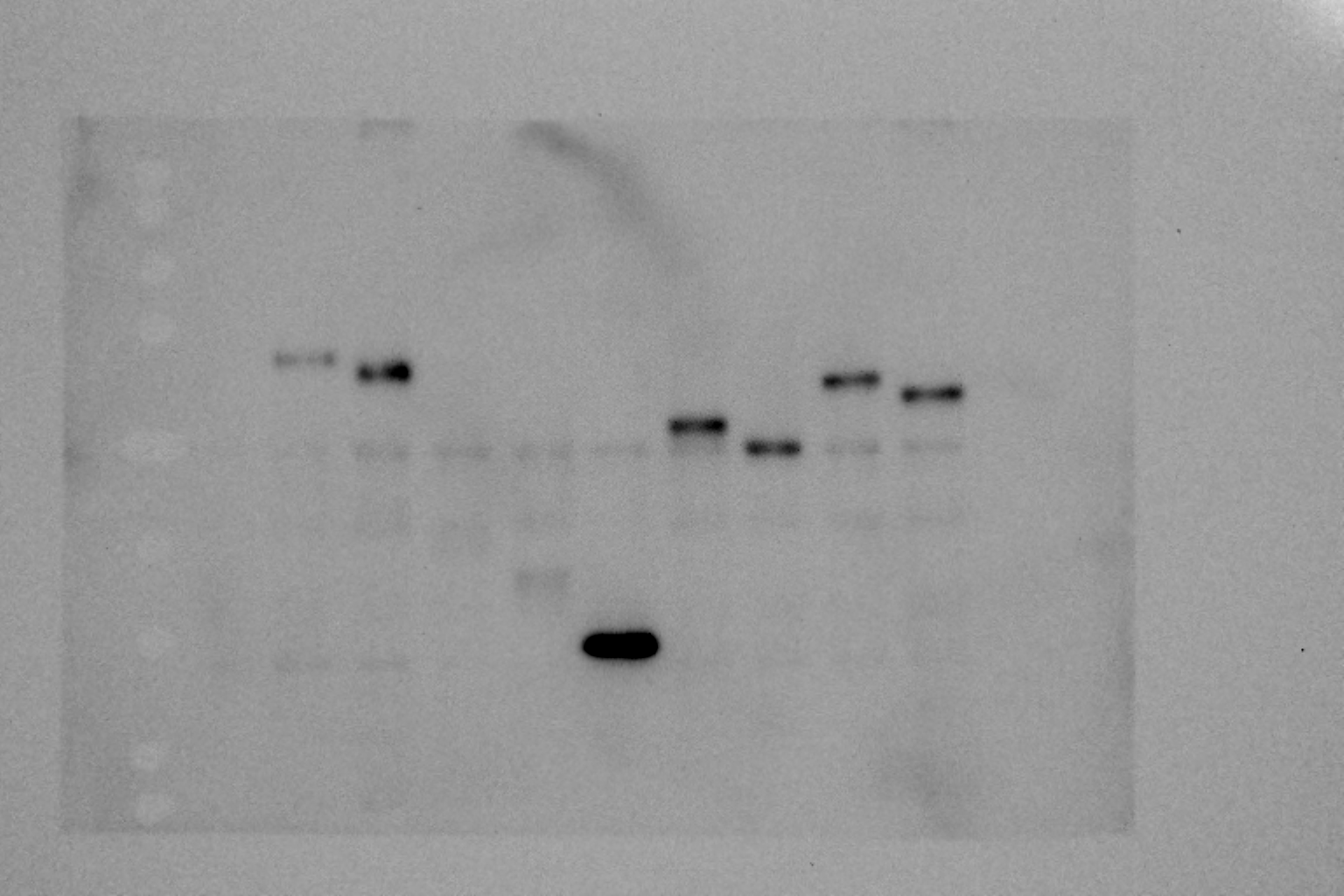

Supplement: Figure 6—source data 1. [file elife-74805-fig6-data1.zip › Fig. 6-source data 1/Fig. 6C Blot IP-Seipin .tif]

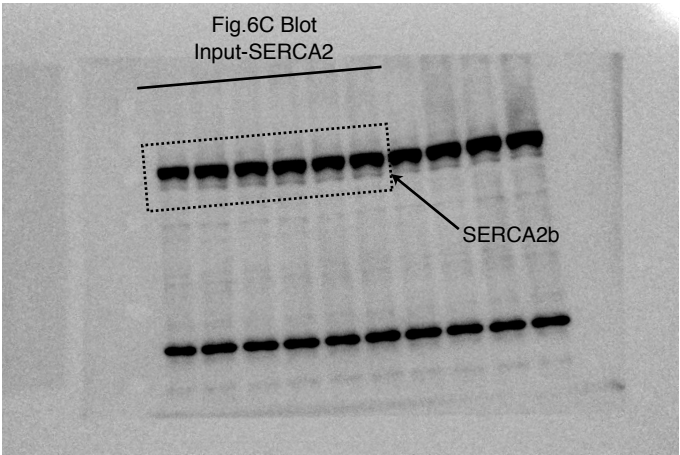

Mouse Anti-SERCA2

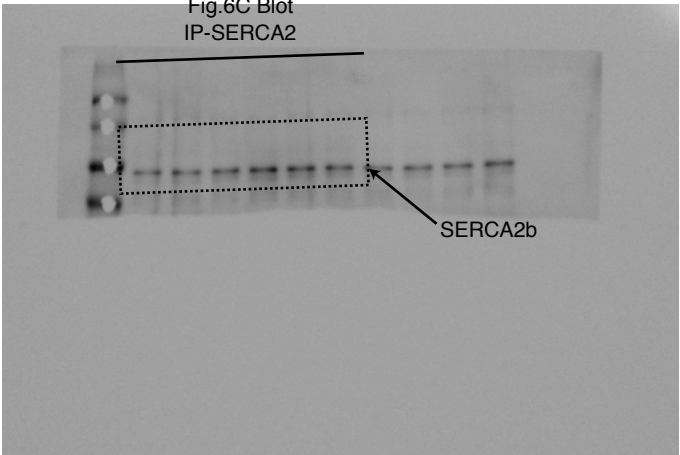

Mouse Anti-SERCA2

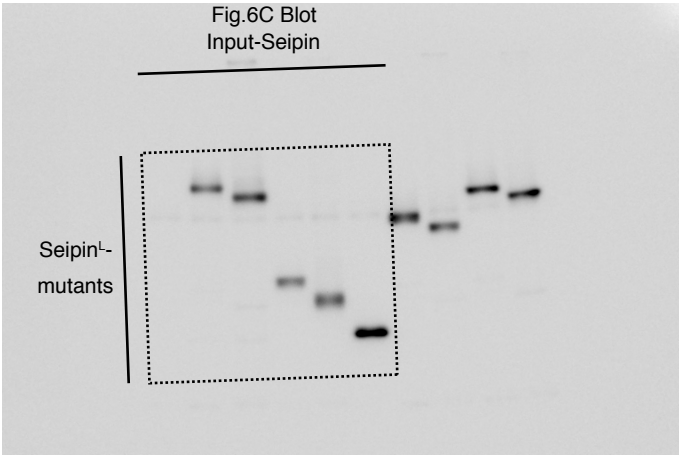

Mouse Anti-Myc Direct

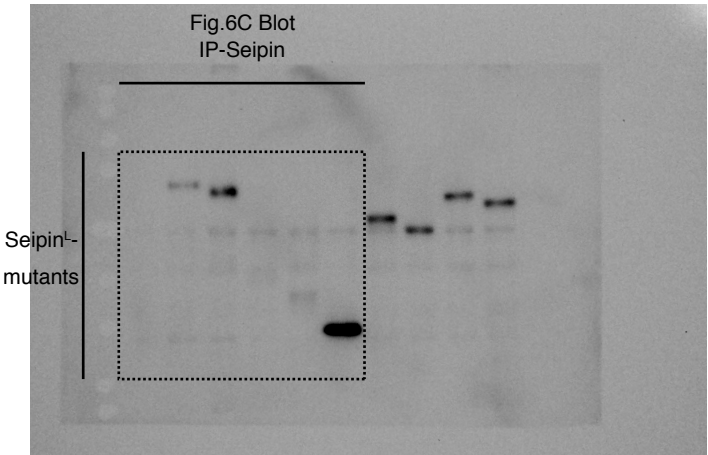

Mouse Anti-Myc Direct

Supplement: Figure 6—source data 1. [file elife-74805-fig6-data1.zip › Fig. 6-source data 1/Fig. 6-source data.pdf]

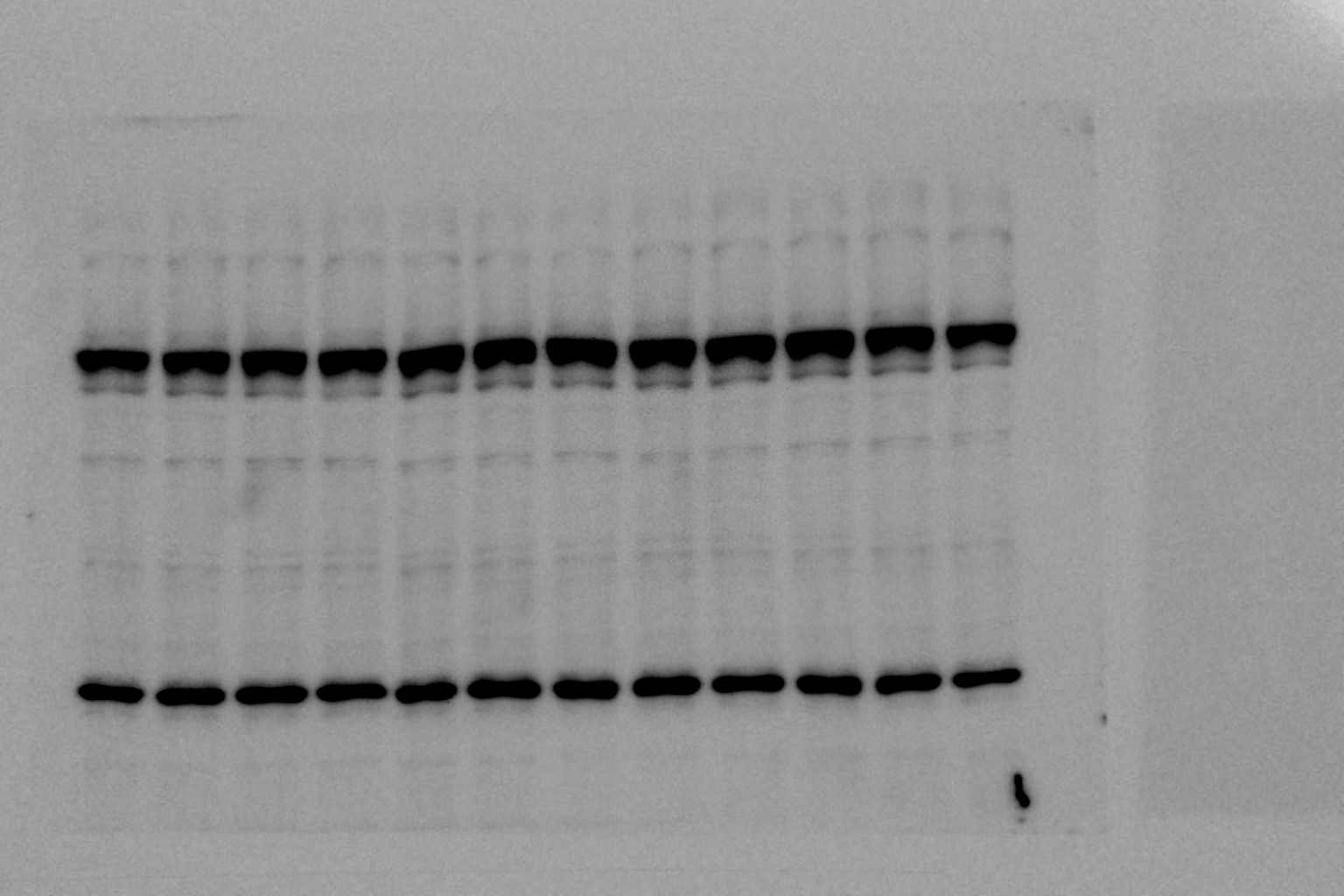

Supplement: Figure 6—figure supplement 1—source data 1. [file elife-74805-fig6-figsupp1-data1.zip › Fig. 6-S1-source data 1/Fig. 6-S1A Blot Input-SERCA2.tif]

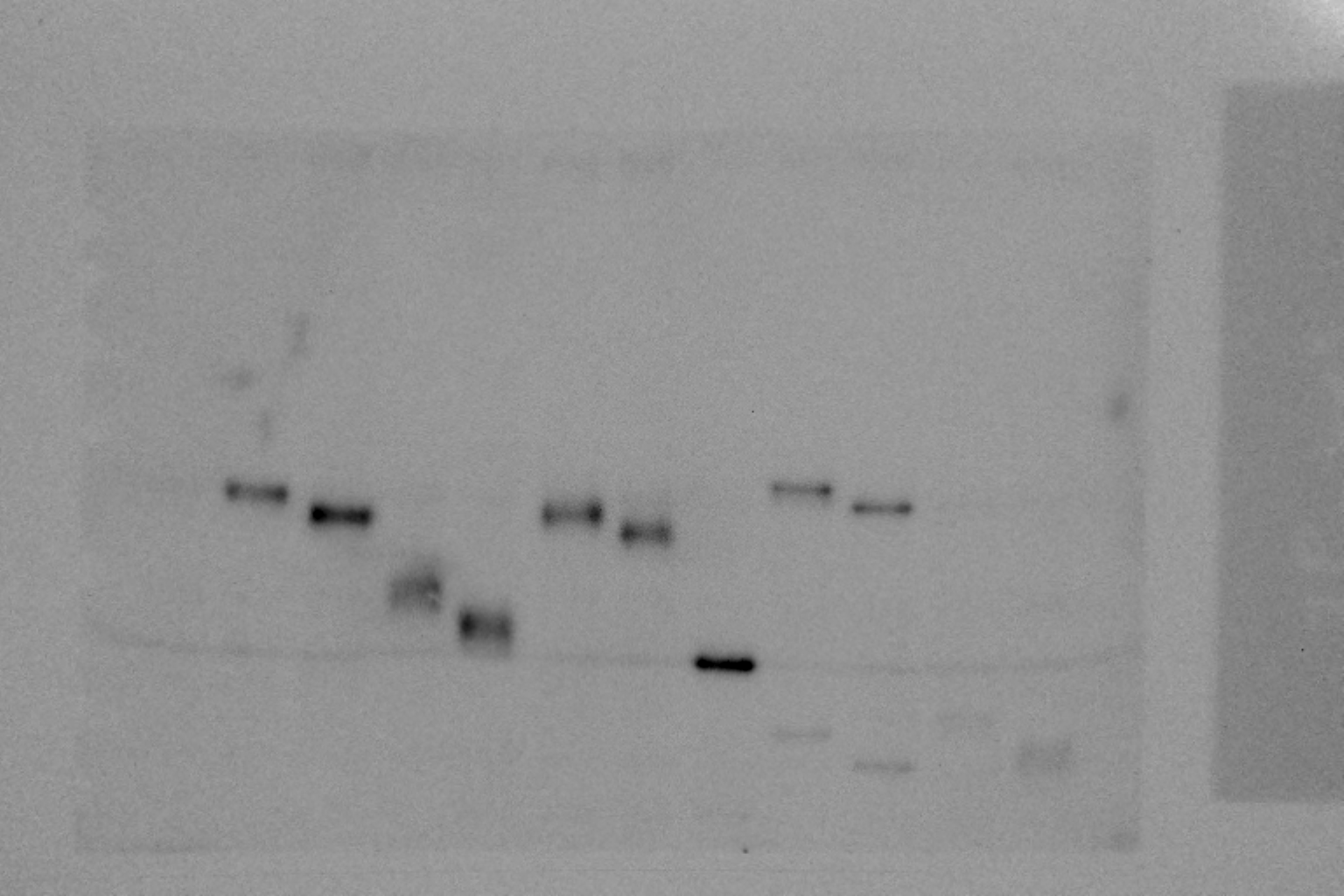

Supplement: Figure 6—figure supplement 1—source data 1. [file elife-74805-fig6-figsupp1-data1.zip › Fig. 6-S1-source data 1/Fig. 6-S1A Blot IP-Seipin.tif]

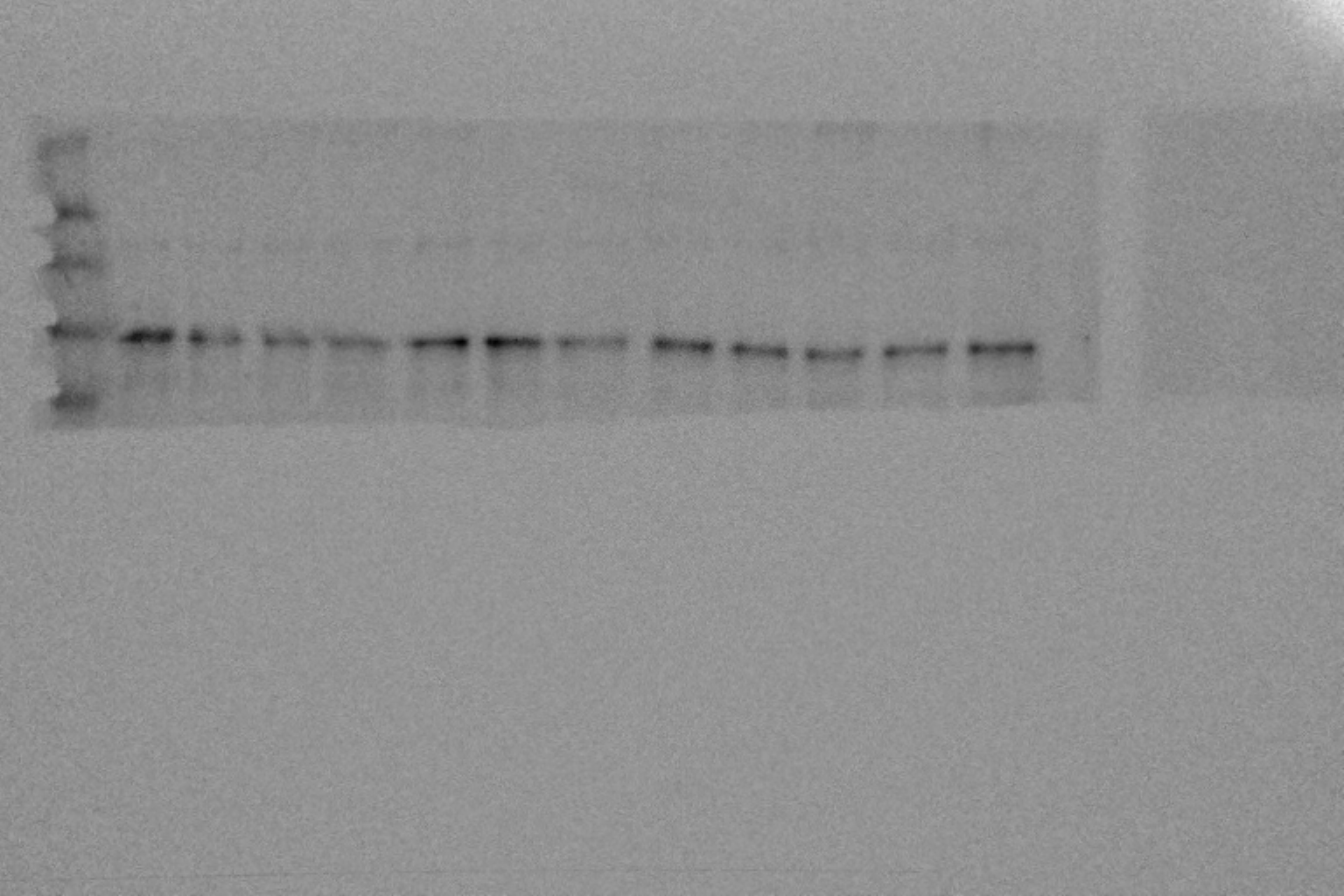

Supplement: Figure 6—figure supplement 1—source data 1. [file elife-74805-fig6-figsupp1-data1.zip › Fig. 6-S1-source data 1/Fig. 6-S1A Blot IP-SERCA2.tif]

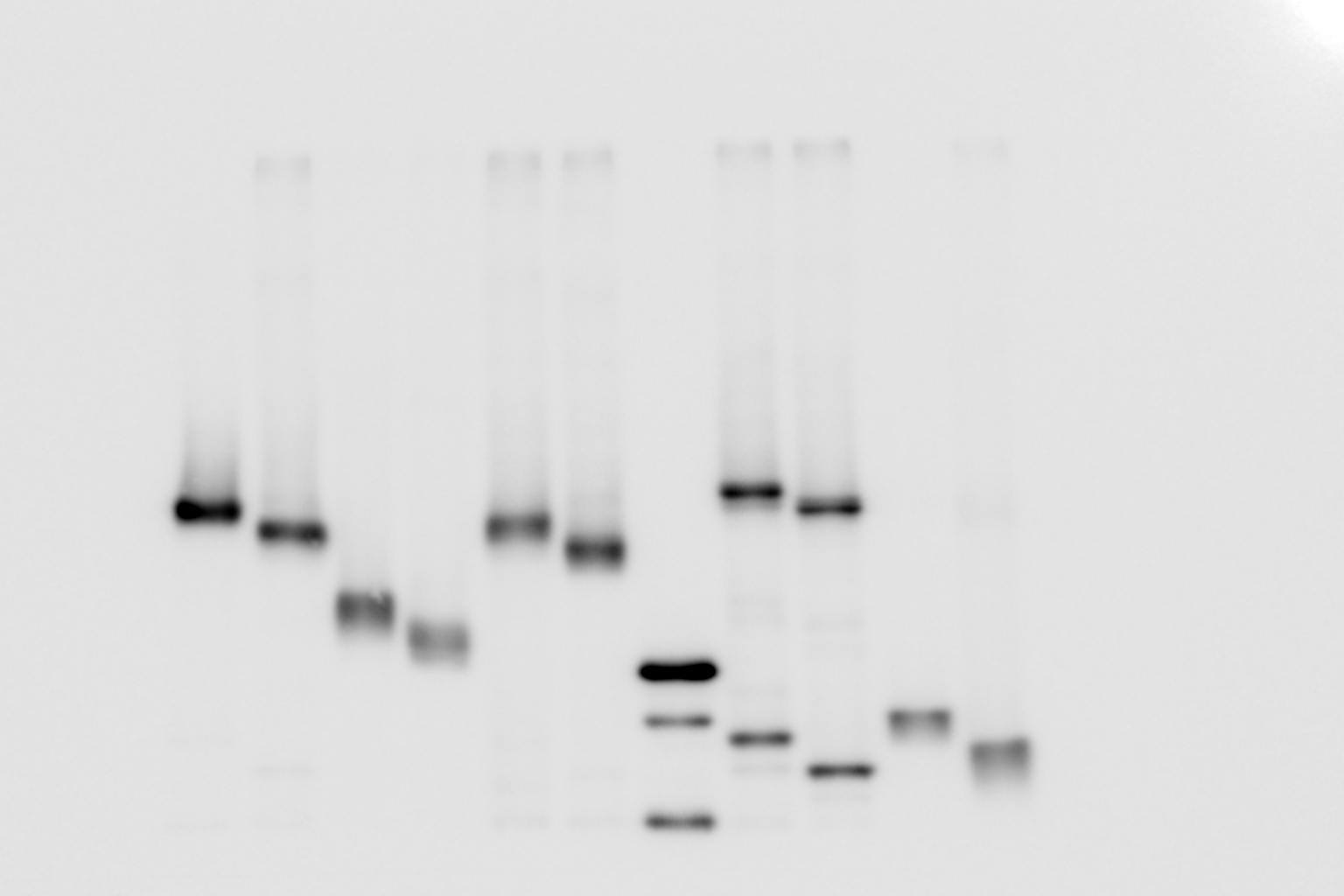

Supplement: Figure 6—figure supplement 1—source data 1. [file elife-74805-fig6-figsupp1-data1.zip › Fig. 6-S1-source data 1/Fig. 6-S1A Blot Input-Seipin .tif]

Fig.6-S1-source data

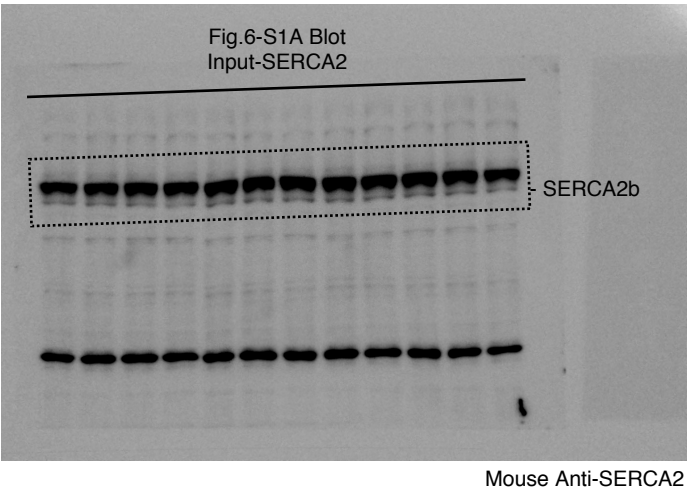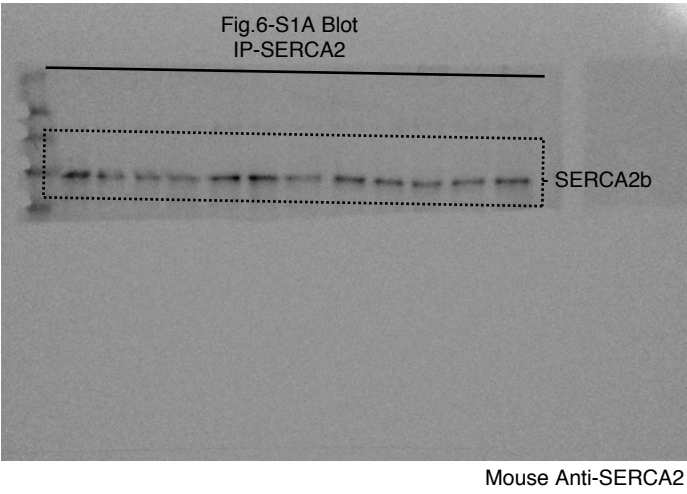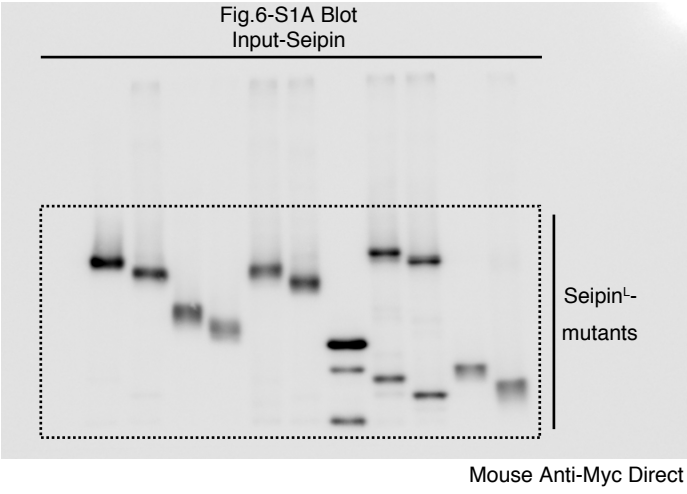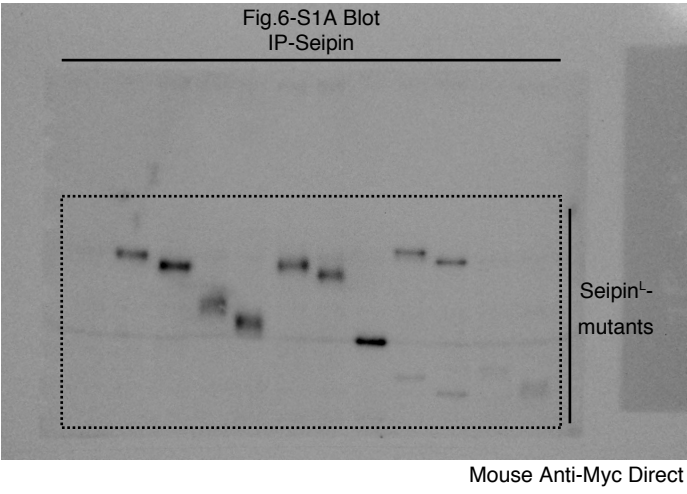

Supplement: Figure 6—figure supplement 1—source data 1. [file elife-74805-fig6-figsupp1-data1.zip › Fig. 6-S1-source data 1/Fig. 6-S1-source data.pdf]

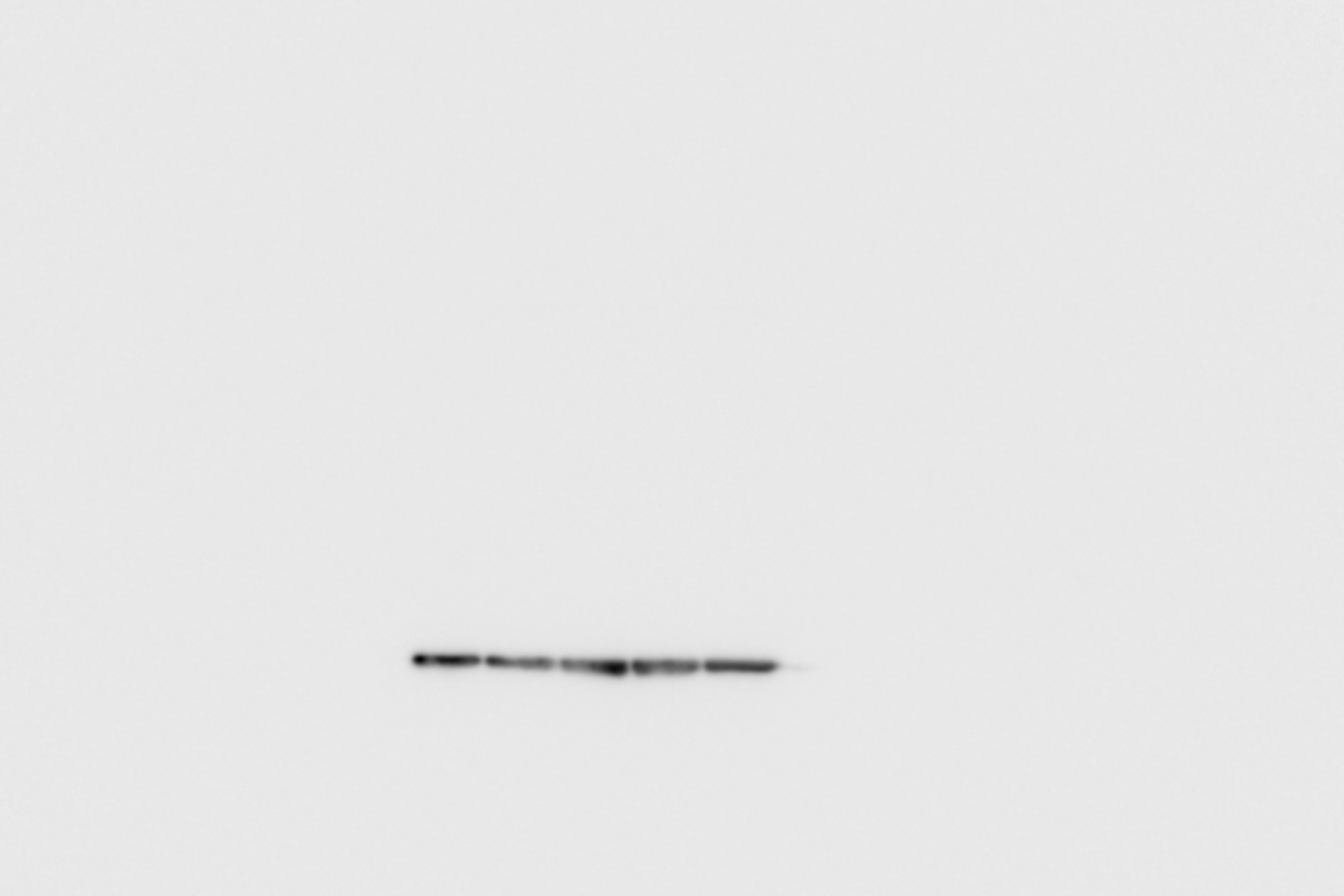

Supplement: Figure 10—source data 1. [file elife-74805-fig10-data1.zip › Fig. 10-source data 1/FIg. 10A(c) Blot GAPDH.tif]

WT + G-CEPIA1er (104 ng/cm<sup>2</sup>)

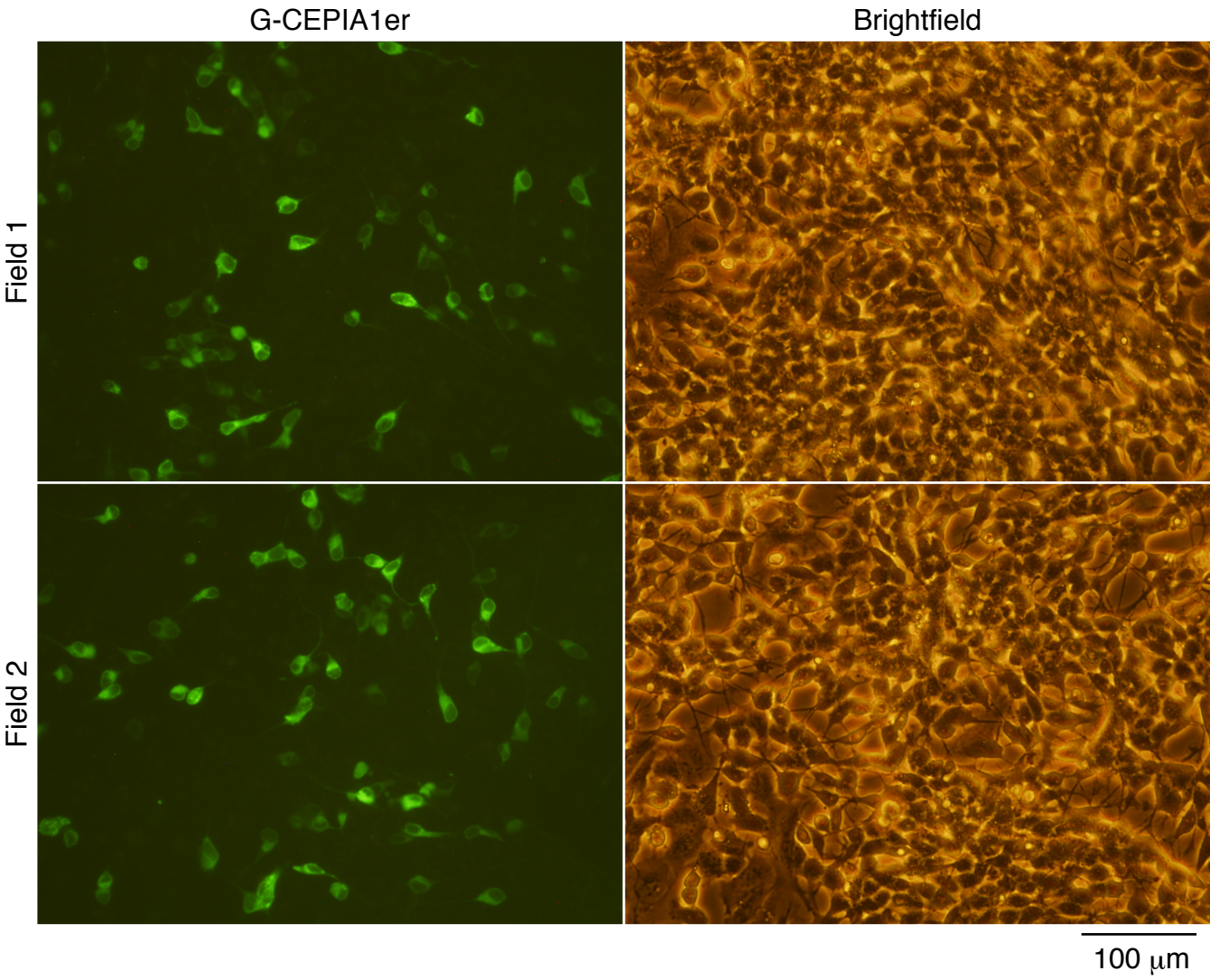

Supplement: Figure 10—source data 1. [file elife-74805-fig10-data1.zip › Fig. 10-source data 1/SH-SY5Y Transfection-efficiency.pdf]

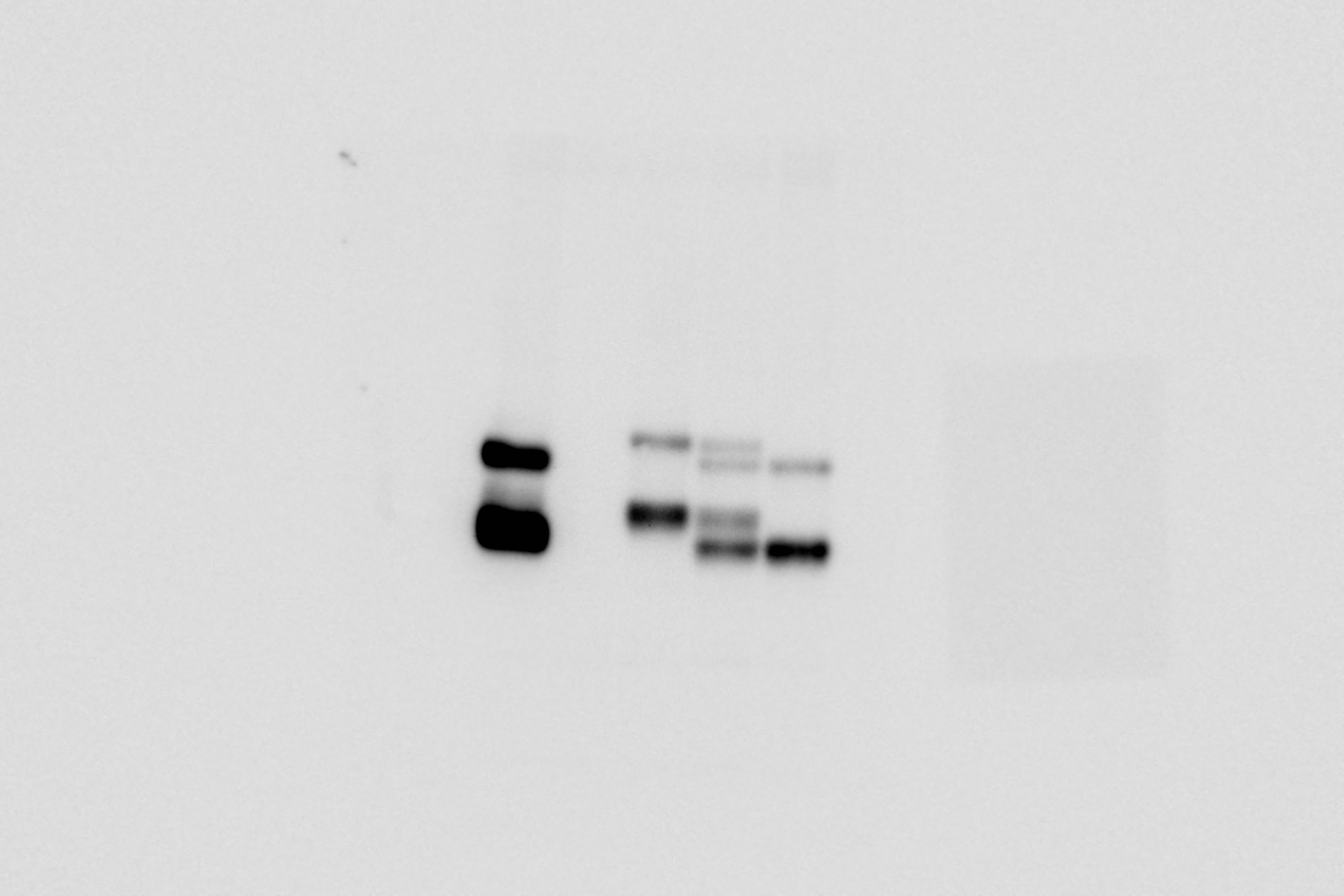

Supplement: Figure 10—source data 1. [file elife-74805-fig10-data1.zip › Fig. 10-source data 1/Fig. 10A(c) Blot Seipin.tif]

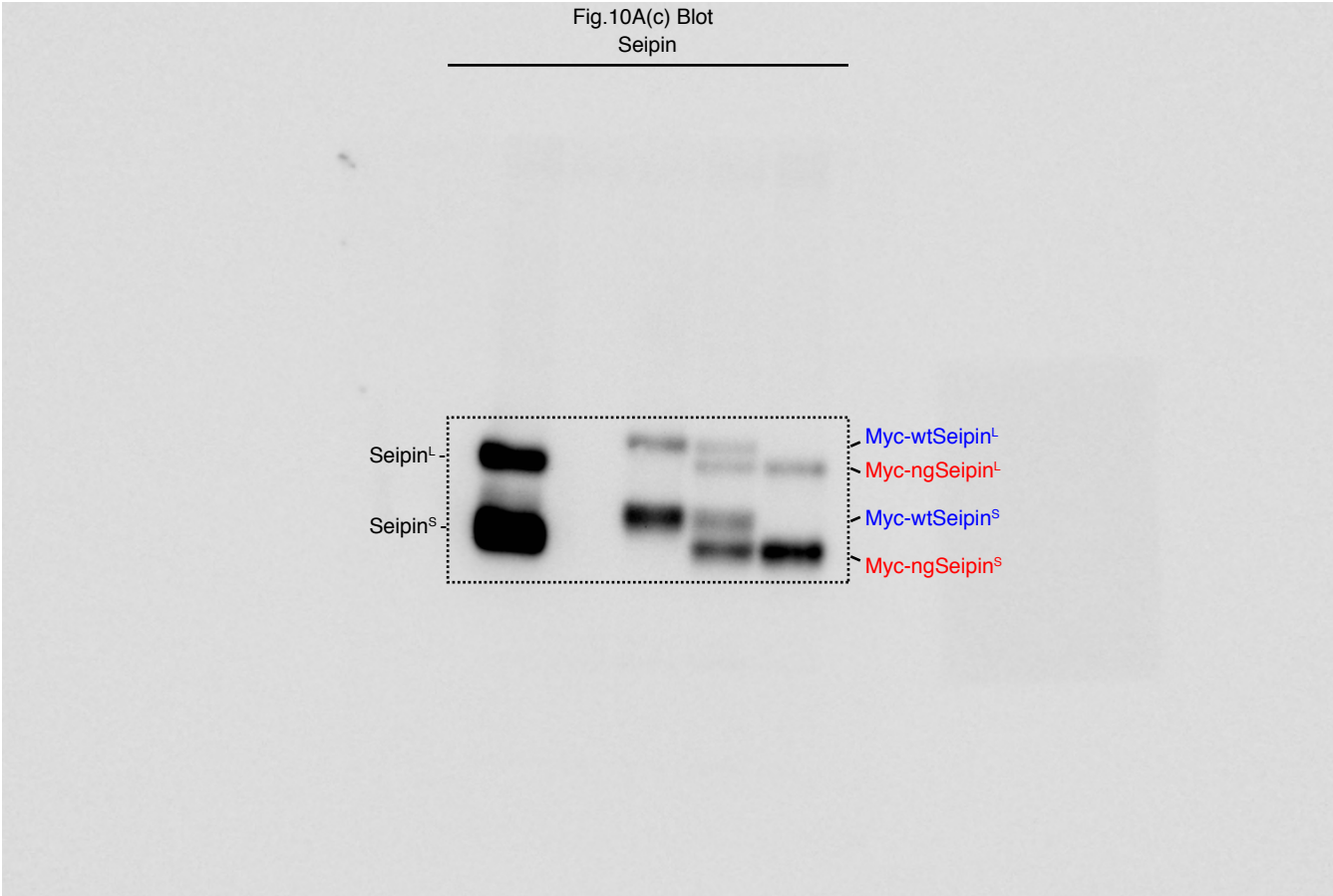

Rabbit anti-Seipin

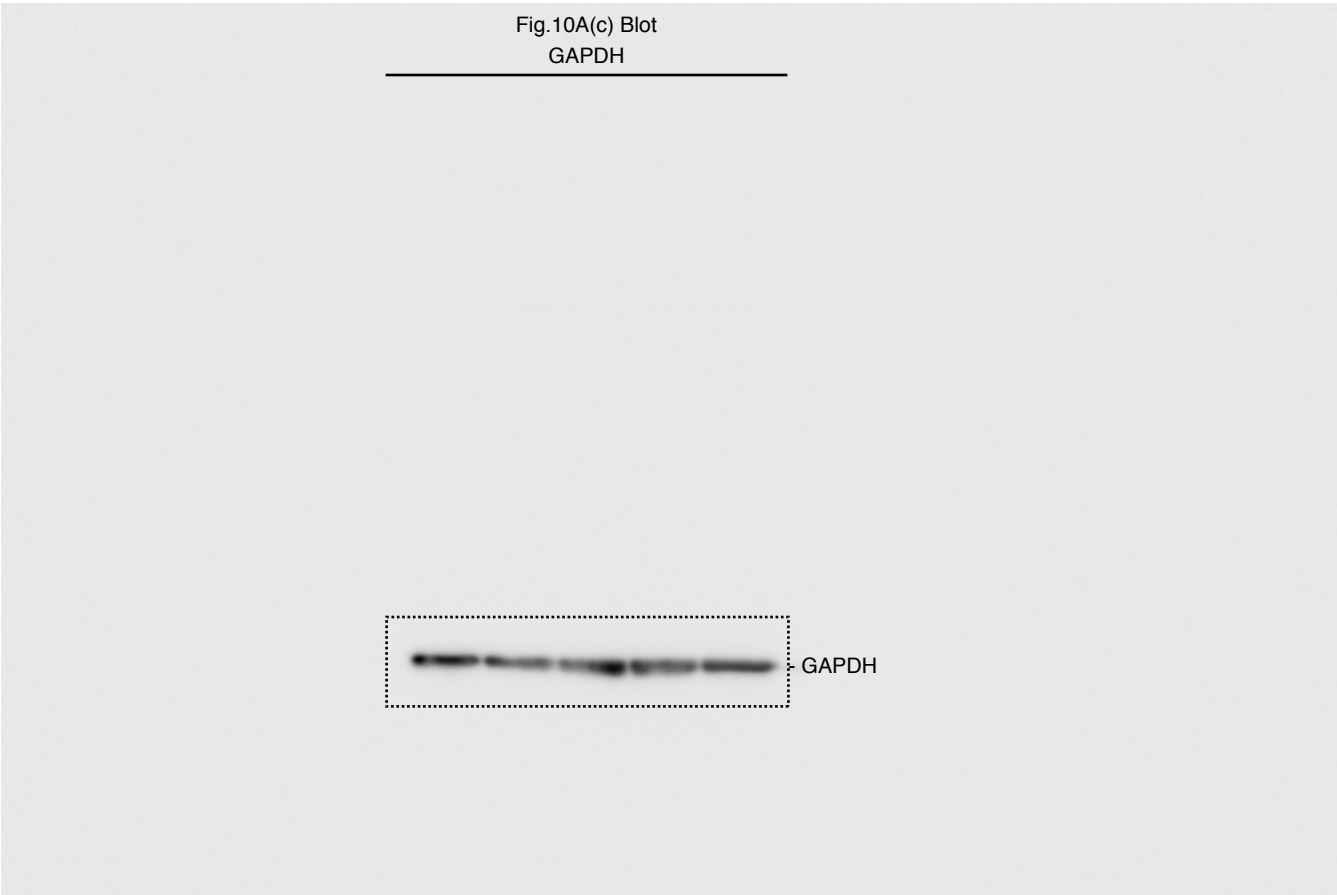

Mouse anti-GAPDH Direct

Supplement: Figure 10—source data 1. [file elife-74805-fig10-data1.zip › Fig. 10-source data 1/Fig.10-source data.pdf]

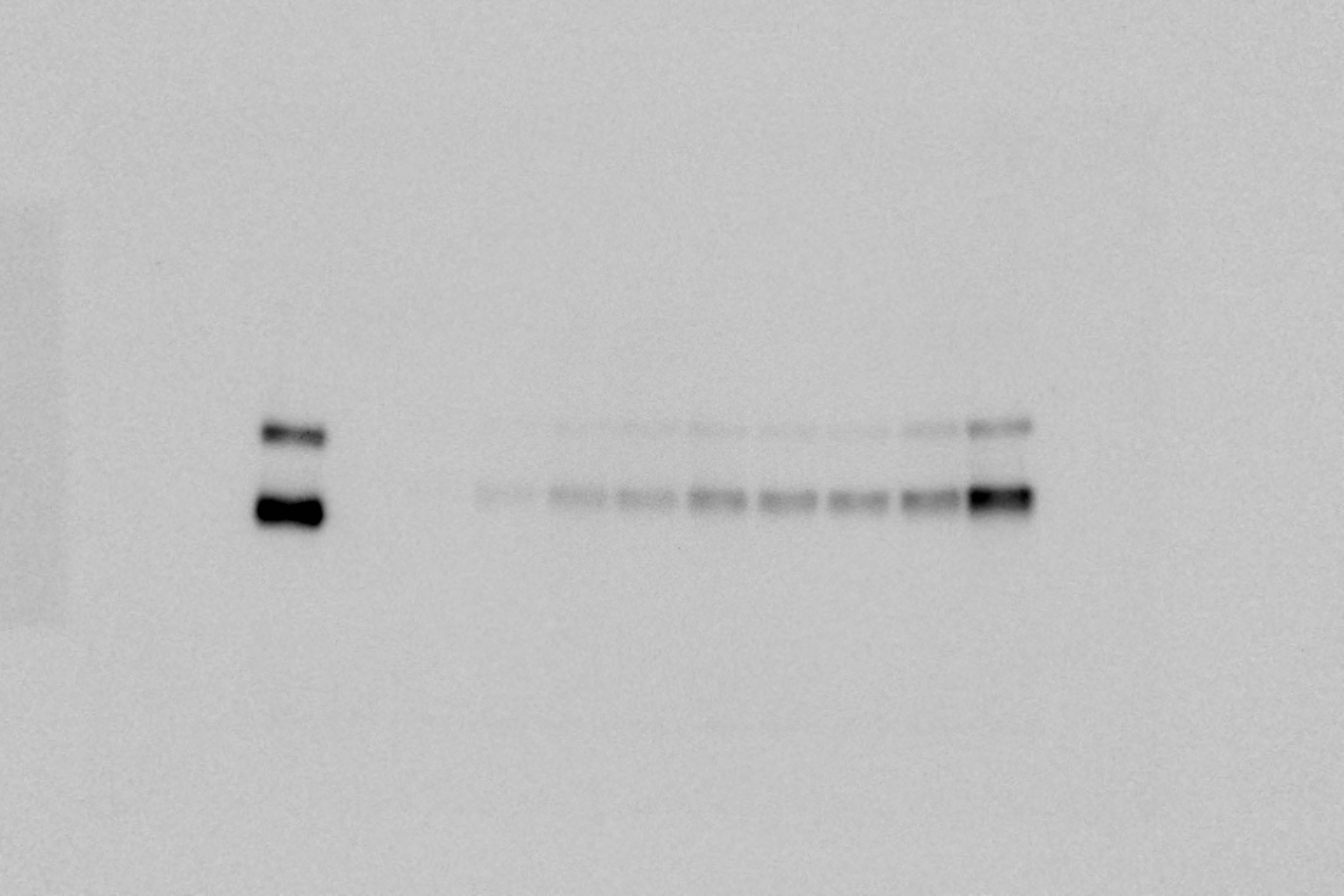

Supplement: Figure 10—figure supplement 1—source data 1. [file elife-74805-fig10-figsupp1-data1.zip › Fig. 10-S1-source data 1/Fig. 10-S1H Blot Seipin.tif]

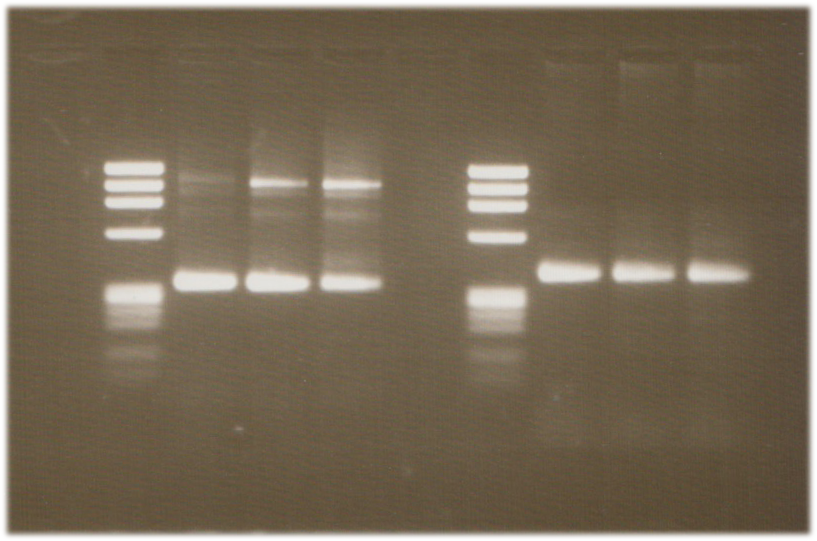

Supplement: Figure 10—figure supplement 1—source data 1. [file elife-74805-fig10-figsupp1-data1.zip › Fig. 10-S1-source data 1/Fig. 10-S1B & S1D Gel.tif]

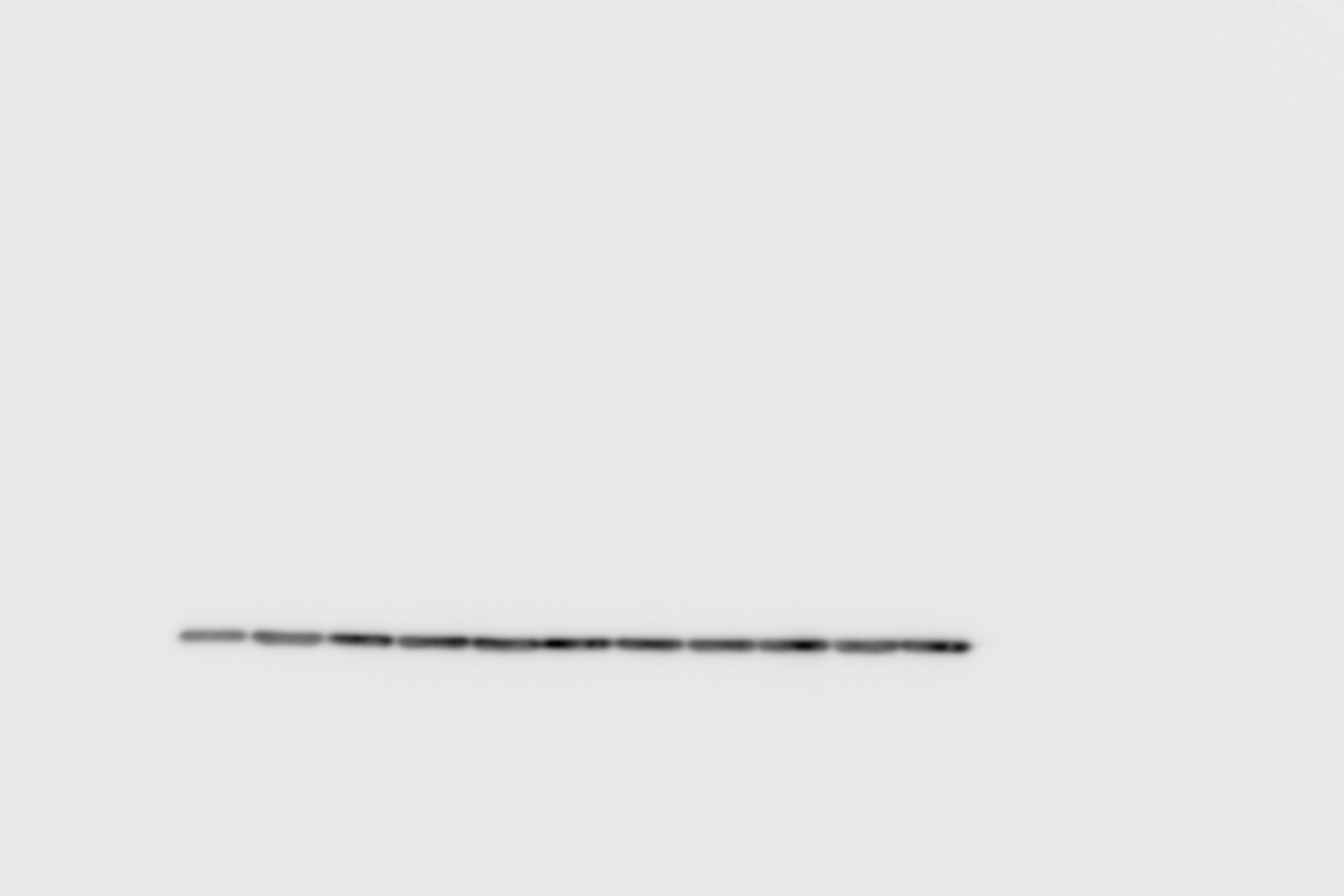

Supplement: Figure 10—figure supplement 1—source data 1. [file elife-74805-fig10-figsupp1-data1.zip › Fig. 10-S1-source data 1/FIg. 10-S1H Blot GAPDH.tif]

Fig.10-S1-source data

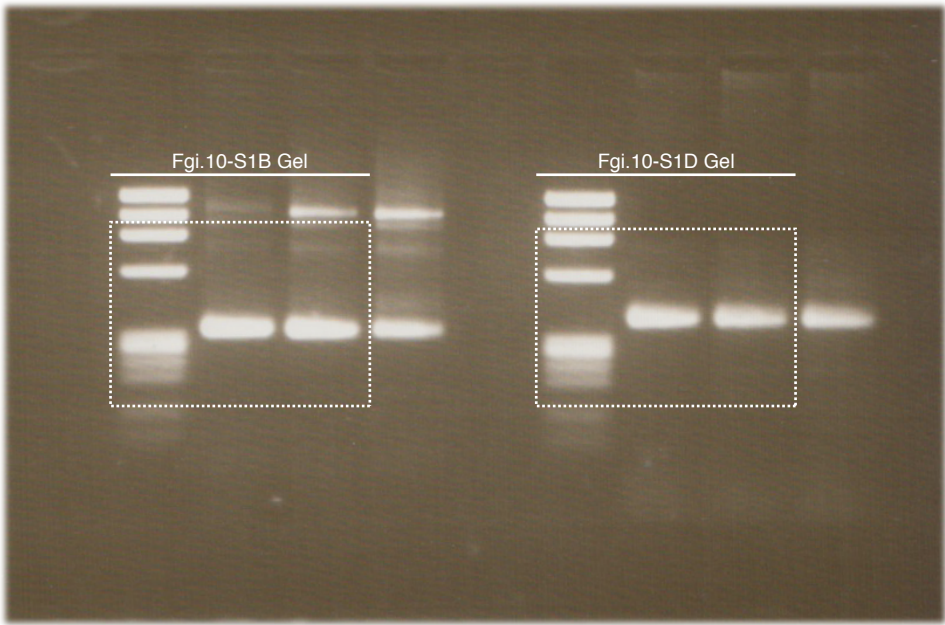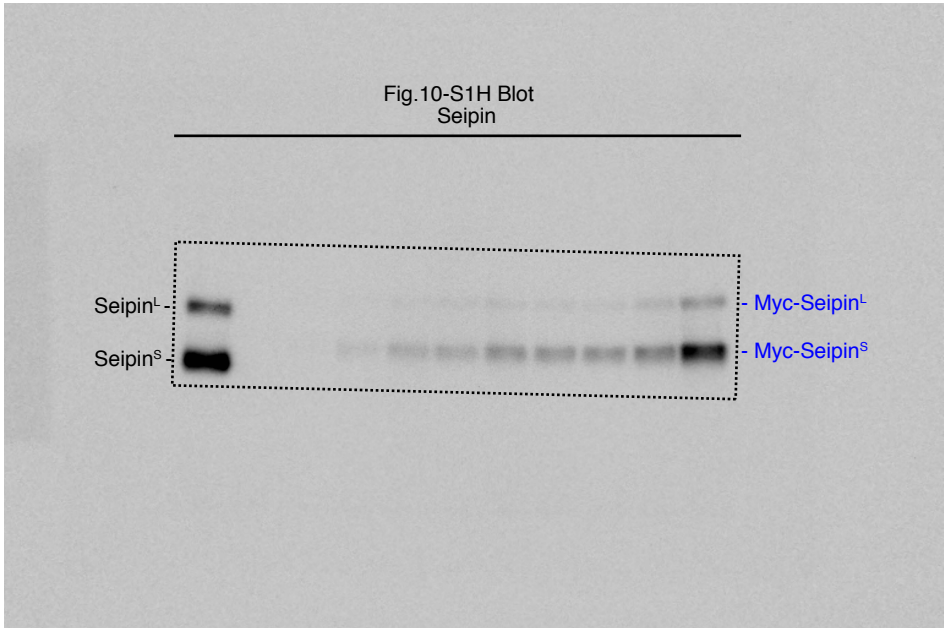

Rabbit anti-Seipin

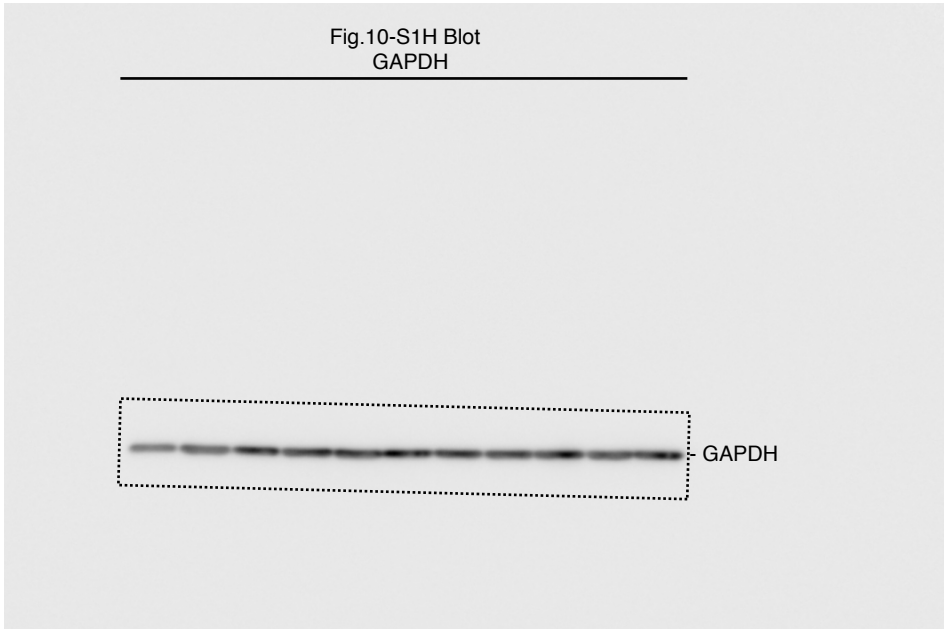

Mouse anti-GAPDH Direct

Supplement: Figure 10—figure supplement 1—source data 1. [file elife-74805-fig10-figsupp1-data1.zip › Fig. 10-S1-source data 1/Fig. 10-S1-source data.pdf]
