# Supplementary material for: A motor neuron disease-associated mutation produces non-glycosylated Seipin that induces ER stress and apoptosis by inactivating SERCA2b
Source: eLife. 2022 Nov 29;11:e74805. doi: 10.7554/eLife.74805 (PMC9708084; doi:10.7554/eLife.74805)
Supplement: Supplementary file 1. [file elife-74805-supp1.docx]

**Supplementary File 1**

| Reagent type or resource | Designation | Source or reference | Identifier | Additional information |
| --- | --- | --- | --- | --- |
| Sequence- based reagent | all del Fw | This paper | Genomic PCR primer | TTTCCATGGTTGCACGATTA |
| Sequence- based reagent | all del Rv | This paper | Genomic PCR primer | AAGCCACATGCAATGGTTTC |
| Sequence- based reagent | inside Fw | This paper | Genomic PCR primer | GGCCTGTGAGCAGAATGTTT |
| Sequence- based reagent | inside Rv | This paper | Genomic PCR primer | GCTGAGGAAGGTGAAGTTGC |
| Sequence- based reagent | Seipin cDNA Fw | This paper | RT-PCR primer | CCTCCTCCTTTCCTCCCTCT |
| Sequence- based reagent | Seipin cDNA Rv | This paper | RT-PCR primer | CTTGCGTTCCTAGCTGCTCT |
| Sequence- based reagent | GAPDH cDNA Fw | Okada et al., 2002 | RT-PCR primer | AGGGCTGCTTTTAACTCTGG |
| Sequence- based reagent | GAPDH cDNA Rv | Okada et al., 2002 | RT-PCR primer | CGTCAAAGGTGGAGGAGTGG |
| Sequence- based reagen | Seipin cDNA Fw2 | This paper | RT-PCR primer | ATGGTCAACGACCCTCCAGT |
| Sequence- based reagen | Seipin cDNA Rv2 | This paper | RT-PCR primer | GACCAAGAACATGCCCAAAT |
| Sequence- based reagent | qBiP Fw | Jin et al., 2020 | qRT-PCR primer | TCTCAGATCTTTTCTACAGCTTCTGA |
| Sequence- based reagent | qBiP Rv | Jin et al., 2020 | qRT-PCR primer | TGTCTTTTGTCAGGGGTCTTTCA |
| Sequence- based reagent | qXBP1 Fw | Jin et al., 2020 | qRT-PCR primer | CTGCTGAGTCCGCATCAGGT |
| Sequence- based reagent | qXBP1 Rv | Jin et al., 2020 | qRT-PCR primer | GAGTCAATACCGCCAGAATCCA |
| Sequence- based reagent | qCHOP Fw | Jin et al., 2020 | qRT-PCR primer | ACCTATGTTTCACCTCCTGGA |
| Sequence- based reagent | qCHOP Rv | Jin et al., 2020 | qRT-PCR primer | CAGTCAGCCAAGCCAGAGAA |
| Sequence- based reagent | qGAPDH Fw | Ninagawa et al., 2014 | qRT-PCR primer | GACCCCTTCATTGACCTCAA |
| Sequence- based reagent | qGAPDH Rv | Ninagawa et al., 2014 | qRT-PCR primer | TTGACGGTGCCATGGAATT |
| Sequence- based reagent | qSERCA1 Fw | This paper | qRT-PCR primer | CCTCACCACCAACCAGATGT |
| Sequence- based reagent | qSERCA1 Rv | This paper | qRT-PCR primer | CCGGTGATGGAGAACTCATT |
| Sequence- based reagent | qSERCA2 Fw | This paper | qRT-PCR primer | GTGCAAATGCCTGCAACTC |
| Sequence- based reagent | qSERCA2 Rv | This paper | qRT-PCR primer | CGACATTGACTTTCTGTCACG |
| Sequence- based reagent | qSERCA3 Fw | This paper | qRT-PCR primer | TCTCGGTGACAGCCGAG |
| Sequence- based reagent | qSERCA3 Rv | This paper | qRT-PCR primer | TCACTCGGGAGCTCGTT |
| Sequence- based reagent | qSERCA2abc Fw | This paper | qRT-PCR primer | TGTCGAACCCTTGCCACTCAT |
| Sequence- based reagent | qSERCA2a Rv | This paper | qRT-PCR primer | GCGGTTACTCCAGTATTGCAGGT |
| Sequence- based reagent | qSERCA2b Rv | This paper | qRT-PCR primer | GCTGCACACACTCTTTACCAGGT |
| Sequence- based reagent | qSERCA2c Rv | This paper | qRT-PCR primer | ACAACTAAAGTTCTGAGCTAAGAACAGGT |
| Sequence- based reagent | qSeipin Fw | This paper | qRT-PCR primer | CCGAGGAGGAGAAACCAGAT |
| Sequence- based reagent | qSeipin Rv | This paper | qRT-PCR primer | CAGGAGCCTGAACCATCACT |
| Sequence- based reagent | qRyR1 Fw | This paper | qRT-PCR primer | GACGAGGTCCAGTTCCTG |
| Sequence- based reagent | qRyR1 Rv | This paper | qRT-PCR primer | TGCTCCTTGAGCACGGT |
| Sequence- based reagent | qIP3R1 Fw | This paper | qRT-PCR primer | GCCAGCTGTCGGAATTAAAG |
| Sequence- based reagent | qIP3R1 Rv | This paper | qRT-PCR primer | GGGTTGACATTCATGTGAGG |
| Sequence- based reagent | qIP3R2 Fw | This paper | qRT-PCR primer | GTGGCTCAAATGATTGTGGA |
| Sequence- based reagent | qIP3R2 Rv | This paper | qRT-PCR primer | CACTGTCGCCTTCATTGCTA |
| Sequence- based reagent | qIP3R3 Fw | This paper | qRT-PCR primer | GTGGATGACCGCTGTGTG |
| Sequence- based reagent | qIP3R3 Rv | This paper | qRT-PCR primer | GCACACCTTGAAGAGGCAGT |
